# Self‐management interventions for children and young people with sickle cell disease: A systematic review

**DOI:** 10.1111/hex.13692

**Published:** 2023-01-03

**Authors:** Brenda A. Poku, Karl Michael Atkin, Sue Kirk

**Affiliations:** ^1^ Division of Nursing, Midwifery and Social Work, School of Health Sciences University of Manchester Manchester UK; ^2^ Department of Sociology University of York York UK

**Keywords:** children, interventions, self‐care, self‐management, sickle cell disease, strategies, young people

## Abstract

**Background:**

Increasing numbers of interventions are being developed to support self‐management for children and young people (CYP) with sickle cell disease (SCD), but no systematic review has systematically synthesized this evidence regarding their characteristics, effectiveness, acceptability and feasibility for all published intervention types.

**Methods:**

The Joanna Briggs Institute guidelines for mixed‐method reviews were followed. A systematic search of eight databases and key journals was conducted from their inception to November 2021. Primary research of self‐management interventions targeting CYP with SCD aged 8–24 years and reporting any health/social outcome and acceptability data were included. Design‐specific standardized critical appraisal instruments were used. Two independent reviewers screened and appraised the articles. A third reviewer resolved disagreements.

**Results:**

Of 1654 articles identified, 38 studies were included. Methodological quality was moderate. Most studies evaluated SCD education, psycho‐behavioural, psychosocial and skills training and/or social support interventions. They appear to demonstrate short‐term improvements in knowledge, social functioning and medical adherence outcomes. Interventions that were multifaceted in content, combined technological platforms and in‐person group‐based formats and involved peers, family and care providers were more acceptable and effective. The long‐term impact of interventions was limited, including CYP's involvement in the intervention development and implementation.

**Conclusions:**

There is inconclusive evidence for any self‐management programme. Nonetheless, support from family, peers and care providers appears to be important for self‐management interventions' effectiveness and acceptability. Future research needs to prioritize CYP involvement in both intervention design and delivery, their wider social context and include CYP with SCD from non‐Black backgrounds.

**Patient and Public Contribution:**

Three young people with SCD recruited acted as the review advisors. They were formally trained in the review process and involved in every aspect of the review: the design, conduct and interpretation of the findings. CYP involvement in the interventions' development and implementation was analysed as part of the review. This systematic review was conducted as part of a wider research project titled: *Understanding fatigue experiences of CYP with SCD to guide the co‐development of a fatigue self‐management intervention*. Two of the young advisors involved in the review were also involved in the development of the project funding application.

## 1 INTRODUCTION

Sickle cell disease (SCD) is globally the most common paediatric genetic blood disorder. Approximately 300,000 babies are born with the condition annually, with a projected 30% increase in incidence by 2050.^1^ Most births occur in Africa, but annually an estimated 2000 and 300 babies are born with SCD in the United States and the United Kingdom, respectively.^2^ Alterations in the shape of red blood cells characterize SCD, leading to blood vessel occlusion and inflammation, infarction, organ damage, pain and profound chronic anaemia.^3^ Consequently, children born with SCD experience comorbidities, have high healthcare needs and reduced life expectancy.^4^, ^5^

With medical advances, mainly in high‐income countries, children born with SCD transition into adulthood. However, SCD is associated with significant medical and psychosocial challenges that often worsen when children and young people (CYP) reach an age to become responsible for managing their condition. As CYP with SCD mature, they have increased decision‐making autonomy and self‐care independence. Self‐management is the purposeful performance of specific learned tasks, activities and behaviours to manage the medical, psychosocial and life impact of a chronic illness.^6^, ^7^, ^8^ SCD self‐management strategies include symptom monitoring, following treatment plans, and ensuring health maintenance practices. Self‐management is crucial as CYP with SCD begin caring for themselves and their illness.

SCD‐related management strategies can negatively impact CYP's perceived quality of life, disrupt school and work attendance and participation and influence their social interactions and relationships.^9^, ^10^, ^11^, ^12^, ^13^, ^14^, ^15^ This may reduce commitment to treatment plans and health maintenance practices.^16^ Effective self‐management support can help mitigate treatment challenges, engage CYP, improve health and social outcomes and reduce illness burden and health costs.^16^, ^17^ Consequently, recent efforts have focused on developing and implementing interventions to support and improve self‐management.

Previous reviews have focused on specific psychological,^18^ medication adherence,^19^ transition programmes^20^ and e‐health interventions,^21^ and assessed their effects on particular outcomes. No systematic review has been identified that focused on all types of self‐management interventions for CYP with SCD and included the full range of health and social outcomes that have been evaluated. This review aimed to: (1) summarize the range and characteristics of self‐management interventions for CYP with SCD; and (2) critically evaluate the effectiveness, acceptability and feasibility of different self‐management interventions, identifying factors influencing their acceptability and implementation.

## 2 METHODS

The Joanna Briggs Institute (JBI) guidelines for conducting mixed‐method reviews^22^ informed the conduct of this review. The review protocol was registered (PROSPERO: CRD42021286422). The review is reported according to the expanded Preferred Reporting Items for Systematic Reviews and Meta‐Analyses (PRISMA) reporting checklist.^23^

### 2.1 Search strategy

First, eight databases: Ovid MEDLINE, EMBASE, PsycINFO and EBM Reviews, ASSIA, CINAHL, Web of Science and Engineering Village were searched from their inception to November 2021. A comprehensive search strategy was developed and modified appropriately across the databases to reflect the various MeSH terms. The following main concepts: ‘child’, ‘young people’, ‘sickle cell disease’, ‘self‐management’, ‘self‐care’ and ‘intervention’ guided the development of the search strategy, with support from a medical reference librarian. See Supporting Information: File 1 for full search strategies applied across the databases. Second, searches of online abstract archives of key paediatric and haematology journals (*Blood*, *Journal of Pediatric Psychology*, *Journal of Pediatric Hematology Oncology, Pediatric Blood Cancer*, *Journal of Adolescent Health*), trial registries (*ISRCTN*, *ClinicalTrials.gov*), review registries (*PROSPERO*, *Cochrane Database of Systematic Reviews, JBI Systematic review Register*) internet‐based resources (*Google*, *Google Scholar*) and OpenGrey from their inception to November 2021 supplemented database searches. Third, reference lists of included studies and systematic review^18^, ^19^, ^20^, ^21^ also provided sources for studies not identified in the database searches. The 3‐step search strategy was implemented by one reviewer (B. A. P.). Authors of recent and ongoing studies were contacted for available publications and/or unpublished data. The review included evidence that met the eligibility criteria detailed in Table 1. The search was unrestricted by country or setting, language or publication year.

**Table 1:** Eligibility criteria

PICOS framework	Inclusion criteria	Exclusion criteria
Population	CYP aged 8–25 years with SCD. The upper age limit is consistent with definitions of young people by the UN, WHO and UK NHS. The lower age limit is informed by the evidence that children with long‐term health conditions may begin to engage (informally) in self‐management around this age.^8^ Papers including samples described as CYP without further specificity around age. Papers involving samples primarily encompassing CYP with a reported mean age between 8 and 25 years. Papers including children and/or young people with long‐term conditions that reported findings for CYP with SCD.	Only included participants over 25 years old. Mean age > 25 years.
Intervention	We defined self‐management as purposeful performance of specific learned tasks, activities and behaviours to manage the medical, psychosocial and life impact of a chronic illness.^6^, ^7^, ^8^ And we defined a self‐management intervention as any intervention, programme or approach designed to develop the ability of and/or support CYP with SCD to manage their long‐term health condition through education, training and support to develop their knowledge, skills or psychosocial resources. All self‐management interventions that target CYP with SCD. All types/forms of self‐management intervention—health, social care and educational interventions—designed to support or facilitate CYP with SCD to take control of and manage their condition, promote their capacity for self‐care and/or maintain and enhance their physical and/or mental health.	Interventions in which children and/or young people are not actively engaged and/or remain passive recipients of knowledge or instructions. Self‐management interventions that target professionals, parents/carers/siblings or families as a whole, without a distinct component(s) for CYP. Papers only describing the development of an intervention. Papers where the effects of the self‐management intervention cannot be distinguished from broader interventions for SCD.
Comparator	Usual care Another self‐management intervention(s) No comparator	
Outcome	No restrictions	
Study design	Primary research, including qualitative, mixed‐methods and quantitative studies of all designs. Evaluations and discursive articles about SCD self‐management interventions. Conference papers that present detailed information about the intervention, research methods and outcome(s).	Dissertations/theses Secondary research (secondary data analysis and literature reviews) Case studies Posters/conference abstracts and proceedings

Abbreviations: CYP, children and young people; SCD, sickle cell disease.

### 2.2 Study selection

All citation records from the database and manual searches were exported into EndNote 20® and deduplicated. Records were independently screened in Covidence® by two reviewers (B. A. P. and S. K.). Any disagreements were adjudicated by the third author (K. M. A.). Reasons for excluding papers were noted.

### 2.3 Quality assessment

Included papers were appraised for methodological quality independently by B. A. P. and S. K. using design‐specific standardized critical appraisal instruments from the JBI System for the Unified Management, Assessment and Review of Information (SUMARI).^24^ For each of the appraisal criteria, the individual appraisers assessed whether the article met each quality criterion (‘yes’), if it failed to meet the criterion (‘no’), if insufficient information was presented to assess the specific criterion adequately (‘unclear’) or if the criterion was not applicable due to the study type, design and/or intervention (‘N/A’) (Tables 3, 4, 5, 6, 7). The total number of appraisal criteria met determined the overall quality of a paper. Papers that met three‐quarters or more of the appraisal criteria were considered to be of high quality, between half and three‐quarters as moderate quality and less than half as poor quality (Tables 3, 4, 5, 6, 7). The quality appraisal did not impact study inclusion or exclusion but was considered in the synthesis.

### 2.4 Data extraction

A typology from previous studies on self‐management interventions guided data extraction on intervention characteristics.^25^, ^26^ Data were extracted on the following dimensions: study aims and objectives, study location, publication year, research design, participant demographics, intervention characteristics and all outcomes measured for effectiveness and acceptability, as well as reported feasibility and implementation issues. For quantitative studies, extracted outcomes comprised descriptive and/or inferential statistical results and themes from the analysis of open‐ended survey questions. For the qualitative studies, data extracted included themes with corresponding quotes. One reviewer (B. A. P.) undertook data extraction, whilst a second reviewer (S. K.) independently extracted data from 10% of all the included papers as a measure to ensure quality control and consistency. Additional information was located and extracted from published protocols/formative papers linked to the included studies. See Supporting Information: File 2 for the detailed data extraction domains.

### 2.5 Data synthesis

Results from the included papers were synthesized narratively in line with the JBI approach. As only one qualitative research paper was included, it was impossible to follow the JBI convergent integrated approach for mixed‐method review. The characteristics of the different interventions were examined and summarized in relation to their effectiveness, acceptability and feasibility for implementation in ‘real life’ settings.

### 2.6 Patient and Public Involvement (PPI)

The review team worked with a PPI group of three young people with SCD recruited from a patient support group. They were trained and involved at every review stage to ensure that patient perspectives (and concerns) were reflected in the review. They commented on the search strategy and the data extraction dimensions developed by the reviewers, helping to identify additional search terms and quality criteria. In addition, they highlighted issues for discussion and recommendations after reviewing the extracted and synthesized data.

## 3 RESULTS

**Figure 1:**
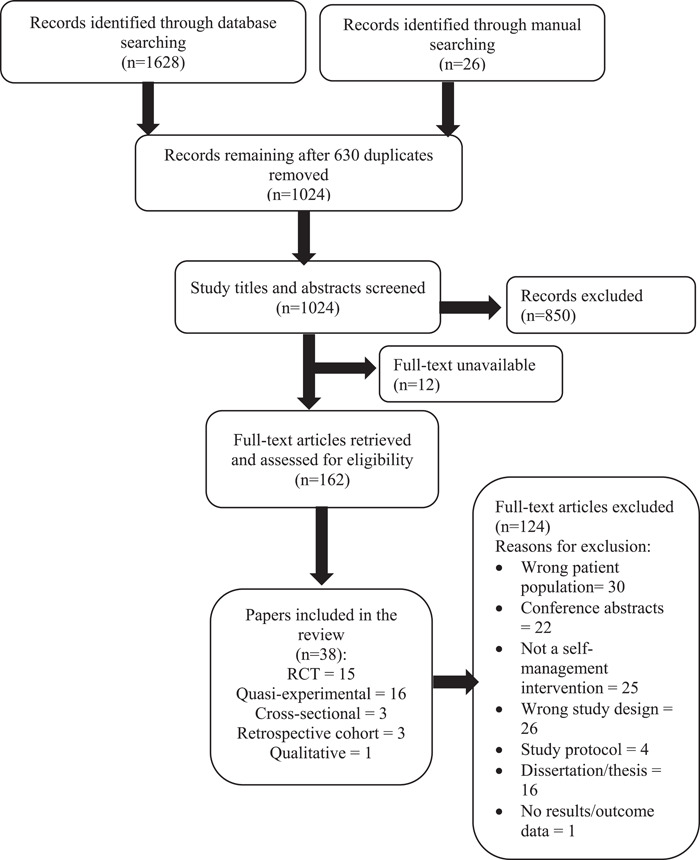
PRISMA flow diagram

A total of 1654 records were imported into EndNote®. After deduplicating the records, 1024 were screened in Covidence®; 38 (representing 32 distinct studies) met all the inclusion criteria and were included in the final review (Figure 1). Of these, 37 were quantitative studies^27^, ^28^, ^29^, ^30^, ^31^, ^32^, ^33^, ^34^, ^35^, ^36^, ^37^, ^38^, ^39^, ^40^, ^41^, ^48^, ^49^, ^50^, ^51^, ^52^, ^53^, ^54^, ^55^, ^56^, ^57^, ^58^, ^59^, ^60^, ^61^, ^62^, ^63^ and 1 was a qualitative study.^64^

### 3.1 Study and sample characteristics

Table 2 presents a summary of the included studies. The 38 articles published between 1987 and 2021 were predominantly from the United States (*n* = 34, 89.5%). More than half (*n* = 26, 68.4%) were published within the last 5 years. There was a preponderance of relatively small‐scale (e.g., single‐site, small‐sample) feasibility/pilot studies (*n* = 21)^28^, ^34^, ^35^, ^36^, ^40^, ^44^, ^46^, ^47^, ^48^, ^49^, ^50^, ^51^, ^55^, ^57^, ^58^, ^60^, ^61^, ^62^, ^63^, ^64^ whose primary objective was to report effectiveness data, acceptability data and/or assess the feasibility and trial design of interventions. The majority (*n* = 31) of the included studies used a randomized^30^, ^31^, ^37^, ^42^, ^43^, ^44^, ^46^, ^47^, ^48^, ^49^, ^50^, ^54^, ^55^, ^58^, ^64^ or non‐randomized comparative study design^27^, ^28^, ^31^, ^32^, ^38^, ^39^, ^41^, ^45^, ^51^, ^52^, ^56^, ^57^, ^59^, ^60^, ^61^, ^62^, ^63^ in which self‐management interventions were either compared with standard care^37^, ^42^, ^45^, ^47^, ^48^, ^49^ or specific control interventions.^30^, ^36^, ^44^, ^46^, ^54^, ^55^, ^58^

**Table 2:** Study and participant characteristics

References	Country	Study design	Target population	CYP participant characteristics (number, mean age, % ethnicity, % gender, % SCD diagnosis)	Intervention name and type	Control	No. of study sites	Attrition rate (follow‐up) (%)
Adegbolagun et al.^28^	Nigeria	Noncontrolled, pre‐post experimental^a^	CYP	*N* = 20; 19.8 years; 100% HbSS; gender and ethnicity not specified	Behavioural	N/A	1	0
Abd Elaziz and Abd Elghany^27^	Egypt	Noncontrolled, pre‐post experimental (full trial)	CYP‐parent dyads	*N* = 100; 12 ± 3.12 years; 55% males; 100% HbSS; ethnicity not specified	Educational, skill training	N/A	2	0
Allemang et al.^29^	Canada	Cohort—retrospective	CYP	*N* = 110; 18.1 years. Gender, diagnosis and ethnicity not specified	Educational, behavioural	N/A	1	14
Barakat et al.^30^	USA	RCT	CYP & family	*N* = 53 (27 IG, 26 CG); 14.24 years IG, 14.10 years CG; 52.9% males IG, 30% males CG; 100% African American IG, 95% African American CG	PAIN (educational, behavioural)	Attention control: DISEASE ED (educational)	1	40
Broome et al.^31^	USA	RCT	CYP	*N* = 67 (35 IG children, 32 IG adolescents); 9.2 years IG children; 15.3 years IG adolescents; 51% males IG children, 65% males IG adolescents; 100% African American. No data provided on CG	Educational, behavioural, social support	Attention control (picnic, trip to a museum)	Not specified	48
Crosby et al.^33^	USA	Cross‐sectional^b^	CYP & parents	*N* = 43; 12.8 ± 3.98 years; 39.5% males; 79% HbSS; 100% African American	Take‐Charge Programme (skill training)	N/A	1	O
Crosby et al.^34^	USA	Cross‐sectional^a^	CYP	*N* = 5; 13–24 years. Gender, diagnosis and ethnicity not specified	iManage (skill training, social support	N/A	1	0
Crosby et al.^35^	USA	Cross‐sectional^a^	CYP	*N* = 22; 18.8 years; 55% males; 100% African American. Diagnosis not specified	Chronic Disease Self‐Management Programme (CDSMP) (educational, skill training)	N/A	1	36
Crosby et al.^36^	USA	RCT^a^	CYP	*N* = 58 (27 IG, 31 CG); 16.7 years IG, 16.3 years CG; 52% females IG, 54% females CG; 46% HbSS IG, 63% HbSS CG; 100% African American	SC Thrive (educational, skill training)	Attention control: SCHealthED (educational)	1	9
Crosby et al.^64^	USA	Qualitative	CYP	*N* = 19; 17.1 years; 58% females; 53% HbSS; 100% African American	SC Thrive (educational, skill training)	N/A	1	N/A
Cozzi et al.^32^	USA	Noncontrolled, pre‐post experimental	CYP	*N* = 8; 10–20 years; 63% females; 88% HbSS; ethnicity not specified	Educational, behavioural	N/A	1	0
Daniel et al.^37^	USA	RCT	CYP & family	*N* = 83; 8.29 years; 50% males; 59% HbSS; ethnicity not specified	Family Taking Control (educational, skill training)	Delayed intervention control	2	27
Dobson and Byrne^38^	USA	Noncontrolled, pre‐post experimental	CYP	*N* = 20; 8.4 years; 60% males; 95% HbSS; 75% African American	Behavioural	N/A	1	0
Dobson^39^	USA	Noncontrolled, pre‐post experimental	CYP	*N* = 20; 8.4 years; 60% males; 95% HbSS; 75% African American	Behavioural	N/A	1	0
Estepp et al.^40^	USA	Cohort—retrospective^a^	CYP	*N* = 83; 13.9 years; 53% females; 91% HbSS; ethnicity not specified	Scheduled Instant Messaging Over the Network (SIMON) (behavioural)	N/A	1	32
Fouda et al.^41^	Egypt	Noncontrolled, pre‐post experimental	CYP	*N* = 50; 14.4 ± 1.15 years; 54% males. Gender and ethnicity not specified	Educational, skill training	N/A	2	0
Gil et al.^42^	USA	RCT	CYP	*N* = 49 (25 IG, 24 CG); 11.9 ± 3 years; 63% males; 67% HbSS; 100% African American	Behavioural	Standard care	1	0
Gil et al.^43^	USA	RCT	CYP	N = 49 (25 IG, 24 CG); 11.9 ± 3 years; 63% males; 67%	Behavioural	Standard care	1	0
Green et al.^44^	USA	RCT^a^	CYP & parents	*N* = 28; 14.3 years; 43% females; 50% Hispanic. IG and CG are not specified.	HABIT (educational, behavioural, skill training)	Two educational brochures	2	11
Hazzard et al.^45^	USA	Noncontrolled, pre‐post experimental	CYP	*N* = 47. Other characteristics not specified	STARBRIGHT World (educational, social support)	Usual education programme	1	0
Hood et al.^46^	USA	RCT^a^	CYP	*N* = 58 (27 IG, 31 CG); 16.7 years IG, 16.3 years CG; 52% females IG, 54% females CG; 46% HbSS IG, 63% HbSS CG; 100% African American	iManage (behavioural, skill training, social support)	Attention control SCHealthED (educational)	1	0
Kaslow et al.^47^	USA	RCT^b^	CYP & family	*N* = 39 (IG = 20, CG = 19); 10.3 years; 62% females; 69% HbSS; 100% African American	Educational, behavioural	Treatment as usual	1	34
Ketchen et al.^48^	USA	RCT^b^	CYP & parents	*N* = 56; 12.7 years IG, 12.18 years CG; 50% males IG, 53% males CG; 100% African American. Diagnosis not specified	STARBRIGHT World (educational, behavioural, social support)	Wait‐list	3	34
McClellan et al.^49^	USA	RCT^a^	CYP & parents	*N* = 19 (9 IG, 10 CG); 13.4 ± 2.9 years; 68% females; 100% African American. Diagnosis not specified	Behavioural	Wait‐list	1	0
Palermo et al.^50^	USA	RCT^a^	CYP & parents	*N* = 25 (15 IG, 10 CG); 14.8 years; 64% females; 64% HbSS; 64% African American	Web‐based Management of Adolescent Pain (Web‐MAP) (behavioural, skill training)	Internet‐delivered pain education	9	20
Phillips et al.^51^	USA	Noncontrolled, pre‐post experimental^a^	CYP & parents	*N* = 60; 7.8 years; 53% males. Diagnosis and ethnicity not specified	Voice Crisis Alert V2 (educational, behavioural, skill training)	N/A	1	20
Rodgers‐Melnick et al.^52^	USA	Noncontrolled, pre‐post experimental	CYP	*N* = 30; 21.04 years; 50% males; 63% HbSS; 100% black ethnicity	Build, Educate, Advance, Transition in Sickle cell disease (BEATS) (educational, behavioural, skill training)	N/A	1	18% attended 0/4 sessions
Saulsberry et al.^53^	USA	Cohort—longitudinal^b^	CYP	*N* = 53; 14 years; 62% males; 66% HbSS; 100% African American	Sickle Cell Transition E‐Learning Programme (STEP) (educational, behavioural)	N/A	1	26
Schatz et al.^54^	USA	RCT	CYP	*N* = 48 (23 IG, 25 CG); 12.3 ± 2.2 years CG, 13.8 ± 2.7 years IG; 43% males CG, 39% males IG; 78% HbSS CG, 74% HbSS IG; 96% African American CG, 91% African American IG	Behavioural	Wait‐list standard care	2	4
Schwartz et al.^55^	USA	RCT^a^	CYP & family	*N* = 49 (25 IG, 24 CG); 14.32 years IG; 56% females IG; 88% HbSS IG; 100% African American	Behavioural	Disease education attention control	1	0
Sil et al.^57^	USA	Noncontrolled, pre‐post experimental^a^	CYP	*N* = 57; 13.33 years; 61% females; 73.7% HbSS; 100 non‐Hispanic black	Comfort Ability Programme for Sickle Cell Pain (CAP for SCP) (educational, behavioural)	N/A	3	53
Sil et al.^56^	USA	Noncontrolled, pre‐post experimental	CYP	*N* = 57; 13.4 years; 56.4% females; 68‐1% HbSS; 98% African American	Behavioural, skill training	N/A	1	38
Smaldone et al.^58^	USA	RCT^a^	CYP & parents	*N* = 28; 14.3 years; 43% females; 50% Hispanic. IG and CG are not specified.	HABIT (educational, behavioural, skill training)	Two educational brochures	2	11
Smith et al.^59^	USA	Noncontrolled, pre‐post experimental	CYP	*N* = 32. Other characteristics not specified	Virginia Commonwealth University Transition Intervention Programme (educational, skill training)	N/A	1	0
Treadwell and Weissman^60^	USA	Noncontrolled, pre‐post experimental^b^	CYP & parents	*N* = 11; 11.6 years; 100% African American. Gender and diagnosis not specified	Desferal Day Camp (educational, behavioural, social support)	N/A	1	0
Viola et al.^61^	USA	Noncontrolled, pre‐post experimental^a^	CYP	*N* = 24; 20.8 ± 2–3 years; 45.8% females; 75% HbSS; 87.5% black or African American	Students Helping Individuals Facilitate Transition (SHIFT) (educational, social support)	N/A	1	0
Wihak et al.^62^	USA	Noncontrolled, pre‐post experimental^a^	CYP & parents	*N* = 8; 10–17 years; 100% black. Diagnosis and gender not specified	Comfort Ability Programme for Sickle Cell Pain (CAP for SCP) (educational, behavioural)	N/A	1	0
Yoon and Godwin^63^	USA	Noncontrolled, pre‐post experimental^b^	CYP	*N* = 22; 10.86 ± 2.58 years; gender, ethnicity and diagnosis not specified	Sickle Cell Slime‐O‐Rama Game (educational)	N/A	1	0

Abbreviations: CG, control group; CYP, children and young people; HbSS, haemoglobin SS genotype; IG, intervention group; *N*, number; N/A, not applicable; RCT, randomised controlled trial; SCD, sickle cell disease.

^a^ Feasibility study.

^b^ Pilot study.

Most studies (*n* = 24) evaluated self‐management interventions targeting CYP, ten targeted CYP‐parent dyads^27^, ^33^, ^44^, ^48^, ^49^, ^50^, ^51^, ^58^, ^60^, ^62^ and four targeted family units.^30^, ^37^, ^47^, ^55^ Most studies (*n* = 23) included self‐selected convenience samples, with sizes ranging from 5^34^ to 110^29^ participants recruited mainly from single sites. Nine studies recruited participants from multiple settings.^27^, ^37^, ^41^, ^44^, ^48^, ^50^, ^54^, ^57^, ^58^ CYP participants were aged between 6 and 24 years, predominantly African American and had homozygous SCD (i.e., HbSS genotype).

### 3.2 Methodological quality of included studies

Overall, the body of evidence was of moderate methodological quality (Tables 3, 4, 5, 6, 7). Several of the randomised controlled trial (RCTs) (*n* = 7) were assessed as being of poor quality. Four were feasibility RCTs with poor reporting quality. Only four RCTs were rated as high quality, three of which were feasibility/pilot RCTs. The RCT studies lacked details of randomization methods, group allocations, blinding of assessors, group comparativeness and statistical analysis methods. The limitations of the quasi‐experimental studies were their short follow‐up length, the multiplicity of outcome measurements and the reliability of the outcome measurement tools used. The cohort and cross‐sectional studies failed to report on how confounding factors were managed in the analysis. Only two quantitative study article^46^, ^64^ sreported sample size calculation or provided reasons for the chosen sample sizes. While all articles reported the number of participants lost to follow‐up, none of the full trials reported conducting an intention‐to‐treat analysis.

**Table 3:** Methodological quality of included RCTs

Quality criterion	Green et al.^44^	Barakat et al.^30^	Broome et al.^31^	Crosby et al.^36^	Daniel et al.^37^	Gil et al.^42^	Gil et al.^43^	Hood et al.^46^	Kaslow et al.^47^	Ketchen et al.^48^	McClellan et al.^49^	Palermo et al.^50^	Schatz et al.^54^	Schwartz et al.^55^	Smaldone et al.^58^
Was randomization used for assignment of participants to treatment groups?	Yes	Unclear	Unclear	Yes	Yes	Unclear	Unclear	Unclear	Unclear	Yes	Unclear	Yes	Yes	Unclear	Unclear
Was allocation to treatment group concealed?	Yes	Unclear	Unclear	Unclear	Yes	Unclear	Unclear	Unclear	Unclear	Yes	Unclear	Yes	Yes	Unclear	Yes
Were treatment groups similar at the baseline?	Unclear	Yes	Unclear	Yes	Yes	Yes	Unclear	Unclear	Yes	Yes	Unclear	Unclear	Unclear	Unclear	No
Were participants blind to treatment assignment?	N/A	N/A	N/A	N/A	N/A	N/A	N/A	N/A	N/A	N/A	N/A	N/A	N/A	N/A	N/A
Were those delivering treatment blind to treatment assignment?	N/A	N/A	N/A	N/A	N/A	N/A	N/A	N/A	N/A	N/A	N/A	N/A	N/A	N/A	N/A
Were outcomes assessors blind to treatment assignment?	Unclear	Unclear	Unclear	Unclear	Unclear	Unclear	Unclear	Unclear	Unclear	Unclear	Unclear	Unclear	Unclear	Unclear	Unclear
Were treatment groups treated identically other than the treatment of interest?	Yes	Unclear	Yes	Yes	Yes	Yes	Yes	Unclear	Yes	N	Unclear	Unclear	Unclear	Unclear	Unclear
Was follow‐up completed and if not, were differences between groups in terms of their follow‐up adequately described and analysed?	Unclear	N	Yes	N	Yes	Unclear	Unclear	Unclear	Yes	Yes	Yes	N	Unclear	N	Unclear
Were participants analysed in the groups to which they were randomized?	Yes	Yes	Yes	Yes	Yes	Yes	Yes	Unclear	Yes	Yes	Unclear	Unclear	Yes	Unclear	Yes
Were outcomes measured in the same way for treatment groups?	Yes	Yes	Yes	Yes	Yes	Yes	Yes	Unclear	Yes	Yes	Unclear	Yes	Yes	Unclear	Yes
Were outcomes measured in a reliable way?	Unclear	N	Yes	Unclear	Unclear	Unclear	Unclear	Yes	Yes	Unclear	Unclear	Yes	Unclear	Unclear	Yes
Was appropriate statistical analysis used?	Yes	Yes	Yes	Yes	Yes	Unclear	Unclear	Yes	Yes	Unclear	Unclear	Yes	Unclear	Unclear	Yes
Was the trial design appropriate, and any deviations from the standard RCT design (individual randomization, parallel groups) accounted for in the conduct and analysis of the trial?	N/A	N/A	N/A	N/A	N/A	N/A	N/A	N/A	N/A	N/A	N/A	N/A	N/A	N/A	N/A
Overall quality	*Moderate*	*Poor*	*Moderate*	*Moderate*	*High*	*Poor*	*Poor*	*Poor*	*High*	*High*	*Poor*	*Poor*	*Moderate*	*Poor*	*High*

Abbreviations: N, number; RCT, randomised controlled trial

**Table 4:** Methodological quality of included quasi‐experimental studies

Quality criterion	Hazzard et al.^54^	Abd Elaziz and Abd Elghany^27^	Adegbolagun et al.^28^	Cozzi et al.^32^	Crosby et al.^34^	Dobson and Byrne^38^	Dobson^39^	Fouda et al.^41^	Phillips et al.^51^	Rodgers‐Melnick et al.^52^	Sil et al.^56^	Sil et al.^57^	Smith et al.^59^	Treadwell and Weissman^60^	Viola et al.^61^	Wihak et al.^62^	Yoon and Godwin^63^
Is it clear in the study what is the ‘cause’ and what is the ‘effect’? (i.e., there is no confusion about which variable comes first)	Yes	Yes	Yes	Yes	Yes	Yes	Yes	Yes	Yes	Yes	Yes	Yes	Unclear	Yes	Yes	Yes	Yes
Were the participants included in any comparison similar?	No	N/A	N/A	N/A	N/A	N/A	N/A	N/A	N/A	Unclear	Yes	N/A	N/A	Yes	N/A	N/A	N/A
Were the participants included in any comparison receiving similar treatment/care, other than the exposure of intervention of interest?	Unclear	N/A	N/A	N/A	N/A	N/A	N/A	N/A	N/A	Unclear	Yes	N/A	N/A	Unclear	N/A	N/A	N/A
Was there a control group?	Yes	No	No	No	No	No	No	No	No	No	No	No	No	No	No	No	No
Were there multiple measurements of the outcome both pre and post the intervention?	No	No	No	Unclear	Yes	No	No	No	No	No	No	No	No	No	No	No	No
Was follow‐up completed and if not, were differences between groups in terms of their follow‐up adequately described and analysed?	Unclear	Yes	Unclear	Unclear	Unclear	Yes	Unclear	Yes	No	No	No	No	Unclear	Unclear	Yes	Unclear	Yes
Were the outcomes of participants included in any comparison measured in the same way?	Yes	N/A	N/A	N/A	N/A	N/A	N/A	Unclear	N/A	Unclear	No	N/A	N/A	Yes	N/A	N/A	N/A
Were outcomes measured in a reliable way?	Unclear	Unclear	Yes	Yes	Yes	Unclear	Unclear	Unclear	Yes	Unclear	Unclear	Yes	No	Unclear	Yes	unclear	No
Was appropriate statistical analysis used?	Yes	Yes	Yes	Yes	Yes	Yes	Unclear	Yes	Yes	Yes	Yes	Yes	Unclear	Yes	Yes	Unclear	Yes
Overall quality	*Moderate*	*Moderate*	*Moderate*	*Moderate*	*High*	*Moderate*	*Poor*	*Moderate*	*Moderate*	*Poor*	*Moderate*	*Moderate*	*Poor*	*Moderate*	*High*	*Poor*	*Moderate*

**Table 5:** Methodological quality of included cohort studies

Quality criterion	Allemang et al.^29^	Estepp et al.^40^	Saulsberry et al.^53^
Were the two groups similar and recruited from the same population?	Yes	N/A	N/A
Were the exposures measured similarly to assign people to both exposed and unexposed groups?	N/A	N/A	N/A
Was the exposure measured in a valid and reliable way?	N/A	No	Yes
Were confounding factors identified?	Unclear	No	No
Were strategies to deal with confounding factors stated?	Unclear	No	No
Were the groups/participants free of the outcome at the start of the study (or at the moment of exposure)?	N/A	Yes	Yes
Were the outcomes measured in a valid and reliable way?	Yes	Unclear	No
Was the follow‐up time reported and sufficient to be long enough for outcomes to occur?	Yes	Yes	Unclear
Was follow‐up completed, and if not, were the reasons to loss to follow‐up described and explored?	Yes	Yes	Yes
Were strategies to address incomplete follow‐up utilized?	N/A	N/A	N/A
Was appropriate statistical analysis used?	Yes	Yes	Yes
Overall quality	*Moderate*	*Moderate*	*Moderate*

**Table 6:** Methodological quality of included cross‐sectional studies

Quality criterion	Crosby et al.^33^	Crosby et al.^34^	Crosby et al.^35^
Were the criteria for inclusion in the sample clearly defined?	Yes	Yes	Yes
Were the study subjects and the setting described in detail?	Yes	Yes	Yes
Was the exposure measured in a valid and reliable way?	Unclear	N/A	Yes
Were objective standard criteria used for measurement of the condition?	Yes	N/A	Yes
Were confounding factors identified?	No	N/A	No
Were strategies to deal with confounding factors stated?	No	N/A	No
Were the outcomes measured in a valid and reliable way?	Unclear	Yes	Unclear
Was appropriate statistical analysis used?	Yes	Yes	Yes
Overall quality	*Moderate*	*High*	*Moderate*

**Table 7:** Methodological quality of included qualitative study

Quality criterion	Crosby et al.^64^
Is there congruity between the stated philosophical perspective and the research methodology?	N/A
Is there congruity between the research methodology and the research questions or objectives?	N/A
Is there congruity between research methodology and the methods used to collect data?	N/A
Is there congruity between the research methodology and the representation and data analysis?	No
Is there congruity between the research methodology and the interpretation of results?	No
Is there a statement locating the researcher culturally or theoretically?	No
Is the influence of the researcher on the research, and vice‐versa addressed?	No
Are participants and their voices adequately represented?	Unclear
Is the research ethical according to current criteria or for recent studies, and is there evidence of ethical approval by an appropriate body?	No
Do the conclusions drawn in the research report flow from the analysis and interpretation of the data?	Unclear
Overall quality	*Poor*

While studies reported differences in the characteristics of those who completed the study and those who dropped out, they failed to report how these had been factored into their analysis. Some RCTs only reported data on the intervention groups. In some studies, not all outcomes were measured for the comparison groups or intervention delivery and the timing of outcome assessments was variable between and within the groups. Several studies assessed outcomes using nonvalidated tools, or there were missing outcome data. Eleven papers reported high follow‐up attrition rates (Table 2). Many studies experienced recruitment challenges, achieving less than 50% of their target sample size. Commonly reported reasons for recruitment difficulties included people's lack of interest in the research, time constraints, inconvenience of intervention format and delivery approaches and perceived lack of intervention benefits.

### 3.3 Description of self‐management interventions

Thirty‐two self‐management interventions were reported in the 38 included articles (Table 8). The majority (*n* = 22) combined different types of self‐management support,^28^, ^29^, ^30^, ^31^, ^32^, ^34^, ^35^, ^36^, ^37^, ^38^, ^39^, ^41^, ^42^, ^43^, ^44^, ^45^, ^51^, ^52^, ^57^, ^60^, ^62^, ^64^ but all had an educational component on general information about SCD or combined this with more specific information about pain, pharmacological treatments, self‐care, clinic appointments, school and academic support, medical insurance and social care supports. Twenty‐eight interventions included skills training programmes (self‐assessment and planning, goal‐setting, problem‐solving, coping and interpersonal communication)^27^, ^28^, ^29^, ^30^, ^31^, ^32^, ^33^, ^34^, ^35^, ^36^, ^37^, ^38^, ^39^, ^41^, ^42^, ^43^, ^44^, ^47^, ^48^, ^49^, ^51^, ^52^, ^53^, ^54^, ^55^, ^56^, ^57^, ^58^, ^59^, ^60^, ^61^, ^64^ and 15 were cognitive‐behavioural therapy programmes.^28^, ^30^, ^31^, ^32^, ^38^, ^39^, ^42^, ^43^, ^49^, ^50^, ^54^, ^55^, ^56^, ^57^, ^62^ Several interventions included elements to facilitate peer and social interactions and support.^34^, ^37^, ^45^, ^46^, ^48^, ^52^, ^57^, ^60^, ^61^ Others^29^, ^33^, ^40^, ^44^, ^58^, ^59^, ^60^, ^61^ focused on promoting medical adherence (i.e., medication adherence and clinic attendance). Several interventions were referred to by names and/or acronyms that reflected their intent or objective(s) (see Table 2).

**Table 8:** Intervention characteristics

References	Intervention content	Theoretical framework	Format/delivery	Duration	Location	Facilitators	Intervention development (PPI)
Abd Elaziz and Abd Elghany^27^	SCD education and self‐care skill training	Not specified	In‐person, group‐based, Face‐to‐face teaching (group discussions, brainstorming, role play, demonstration & redemonstration), printed materials	3 daily sessions, 20–30 min/session	Outpatient	Research staff (nursing background)	Developed by researchers with no PPI or CYP involvement.
Adegbolagun et al.^28^	SCD education, pain management, skill training (activity scheduling, relaxation techniques, attention diversion, effective communication), general self‐management,	Not specified	In‐person, group‐based, Face‐to‐face teaching	5 weekly sessions. Duration of each session not indicated	Outpatient	Research staff (psychology background)	Adapted from a previous study. Adaptation done by researchers with no PPI.
Allemang et al.^29^	SCD education, self‐management counselling (medication appointment adherence), health and social system navigation support	Model for improvement (a quality improvement model)	Face‐to‐face teaching, telephone and e‐mail support, printed resources	120–180 min/in‐person sessions. The overall duration was 12 months	Outpatient, remote (phone call, e‐mail)	Transition navigator (social worker)	Developed based on survey on the facilitators and barrier to successful transition from young people with SCD. Programme content and format developed with input from clinicians. No PPI or CYP involvement.
Barakat et al.^30^	Knowledge and skill training for pain management (relaxation, guided imagery, positive coping self‐statements)	Not specified	In‐person, family‐based, Face‐to‐face teaching, digital resources (guided imagery audiotapes), phone calls	4 sessions, the first 3 sessions were 2 weeks apart with a booster session 1 month apart. 90 min/sessions	Home, phone calls	Psychology students & psychologists	Not specified
Broome et al.^31^	SCD education, cognitive‐behavioural skill training (relaxation, distraction breathing, guided imagery), art therapy (drawing, murals, art, interaction) to express feelings about pain and develop social skills	Not specified	In‐person, group‐based, Face‐to‐face teaching, printed materials, digital resources (videotapes, audiotapes)	4 sessions over 6 weeks. Duration of each session not indicated.	Outpatient	Clinical nurse specialists, art therapist	No PPI or CYP involvement.
Crosby et al.^33^	Self‐assessment of barriers to treatment adherence (medication and appointments), action planning, problem‐solving skills	Not specified	Voice automated interactive web‐based assessment tool	Not specified	Remote (web‐based)	Self‐guided	CYP with SCD and their parents involved in the second phase (identification of content domains) of development.
Crosby et al.^34^	Pain monitoring/tracking, skill training (goal setting, goal evaluation), social support (group formation and competition)	Not specified	Digital, Mobile app, individual‐based	6 weeks	Web‐based (digital)	Self‐directed	3‐phased development process (survey, design and usability testing). CYP involved in all the development phases
Crosby et al.^35^	SCD education, action planning and problem‐solving skill training	Not specified	In‐person, group‐based, face‐to‐face teaching	6 weekly sessions, 150 min/session	Outpatient	Lay leaders	Pre‐existing programme (Stanford Chronic Disease Self‐Management Programme) without adaptation for CYP with SCD.
Crosby et al.^36^	SCD education, skills training (communication, problem‐solving, pain, mood, mind and health management)	Not specified	In‐person, group‐based and remote (Zoom & mobile app) face‐to‐face teaching (brainstorming, video vignettes, modeling, role‐play, rehearsal, group exercises, group discussions), e‐handouts	6 weekly sessions (first 3 in‐person and last 3 via Zoom) plus optional booster session after 2 weeks, 90 min/session	Outpatient, web‐based	Psychology student, psychologist	3‐phased development process (survey, design and usability testing). CYP involved in all the development phases.
Crosby et al.^64^	SCD education, skills training (communication, problem‐solving, pain, mood, mind and health management)	Not specified	In‐person, group‐based, face‐to‐face teaching (brainstorming, video vignettes, modelling, role‐play, rehearsal, group exercises, group discussions), e‐handouts, web‐based (individual‐based)	6 weekly sessions (first 3 in‐person & last 3 via Zoom) plus optional booster session, 90 min/session	Outpatient, web‐based	Psychology student, psychologist	3‐phased development process. (survey, design and usability testing). CYP involved in all the development phases.
Cozzi et al.^32^	behavioural skill training for pain management (breathing techniques, electromyographic and thermal biofeedback)	Not specified	In‐person, individual‐based, telephone‐based, Face‐to‐face teaching, audiotapes and phone calls	13 weekly sessions, 20–30 min/session	Outpatient, home	Clinical psychologist and prerecorded audiotapes	Pre‐existing intervention adapted for CYP with SCD but no PPI or CYP involvement in the adaptation process.
Daniel et al.^37^	SCD information, school support information, problem‐identification and problem‐solving skills	Problem‐solving	Face‐to‐face (role‐play, problem‐solving) telephone teaching	4 weekly sessions plus 3 booster phone call sessions. 75–90 min/in‐person session & 30 min/booster session	Outpatient, telephone	Psychology students	Intervention materials were developed based on focus group feedback and reviewed by a focus group of parents and CYP with SCD.
Dobson and Byrne^38^	guided imagery education and skill training	Cognitive‐behavioural theory & Bandura self‐efficacy theory	In‐person, individual‐based, Face‐to‐face teaching, audiotapes	One session, 15–45 min	Outpatient	Child life specialist	Not specified.
Dobson^39^	guided imagery education and skill training	Cognitive‐behavioural theory & Bandura self‐efficacy theory	In‐person, individual‐based, Face‐to‐face teaching, audiotapes	One session, 15–45 min	Outpatient	Child life specialist	Not specified.
Estepp et al.^40^	Daily text reminders about taking hydroxyurea. Participants can reply to the system	Not specified	Telephone‐based, individual‐based	12 months	Remote (telephone‐based)	Automated	Text message content customized for each CYP participant. No PPI or CYP involvement in the intervention development. Intervention developed by clinical informatics staff.
Fouda et al.^41^	SCD education, self‐care practices/skill training	Not specified	Face‐to‐face teaching (group discussion, role‐play), printed materials	6 sessions over 3 days, 30–60 min/session	Outpatient	Research staff (nursing background)	Developed by researchers based on the assessed needs of CYP with SCD. No PPI or CYP involvement.
Gil et al.^42^	Cognitive behavioural skill training for coping with pain (breathing, relaxation, guided imagery, calming self‐statements)	Not specified	Face‐to‐face, individual‐based, in‐person teaching, prerecorded audiotapes for self‐directed practice, homework assignments, phone calls	2 sessions. First session 45 min, second session a week later to review skills taught/learned	Outpatient, remote (telephone)	Research staff (psychology background) plus self‐directed practice for 1 week	Modelled after one used in an SCD adult population. PPI or CYP involvement not specified.
Gil et al.^43^	Cognitive behavioural skill training for coping with pain (breathing, relaxation, guided imagery, calming self‐statements)	Not specified	Face‐to‐face, individual‐based, in‐person teaching, prerecorded audiotapes for self‐directed practice, homework assignments, phone calls	In‐person session—45 min. Weekly telephone contacts for 4 weeks	Outpatient, remote (telephone)	Research staff (psychology background) plus self‐directed practice for 1 month	Modelled after one used in an SCD adult population. PPI or CYP involvement not specified.
Green et al.^44^	SCD and hydroxyurea education, adherence barriers identification, action planning, problem‐solving skills, automated medication text reminders	Not specified	Face‐to‐face teaching, text messages, phone calls, printed brochures	5 sessions, 60–90 min/session	Outpatient, remote	Community health workers	Not specified.
Hazzard et al.^45^	SCD education, recreational activities (games, arts, crafts) to enhance coping and social support		Face‐to‐face teaching, web‐based (video conferencing, chatrooms, e‐games, arts, crafts	Up to the time of hospital discharge	In‐patient, web‐based	self‐guided. Healthcare professionals present educational activity (15–45 min).	Pre‐existing programme with the educational component adapted for CYP with SCD. Adapted component reviewed by a team of clinicians. No PPI or CYP involvement in the adaptation process.
Hood et al.^46^	Pain monitoring/tracking, skill training (goal setting, goal evaluation), social support (group formation and competition)	Not specified	Digital, Mobile app, individual‐based	6 weeks	Web‐based (digital)	Self‐directed	3‐phased development process (survey, design and usability testing). CYP involved in all the development phases.
Kaslow et al.^47^	SCD education, pain management techniques, problem‐based coping practices	Stress‐coping‐adjustment framework	In‐person, family‐focused, Face‐to‐face teaching (role‐play, games), homework, printed materials	6 weekly sessions, 60 min/session	Outpatient	Psychology students	Input from clinicians and a SCD family member. No CYP involvement.
Ketchen et al.^48^	SCD education, skill training (interpersonal communication, self‐expression, distraction‐effective elevation)	Not specified	Web‐based (video conferencing, chatrooms, e‐recreational activities), a CD‐ROM game, homework	6 weeks intervention	In‐patient, home, web‐based	Self‐guided. Weekly reminder phone calls from the research team	Pre‐existing programme used without adaption for CYP with SCD. Programme development not identified.
McClellan et al.^49^	Cognitive‐behavioural coping skills training for pain management (deep breathing, guided imagery, relaxation)	Not specified	In‐person, family‐based, face‐to‐face teaching (participatory activities and demonstrations), printed materials, e‐modules, telephone calls, e‐mails	One 120 min in‐person session plus digital engagement over 2 months and weekly telephone calls within the month	Outpatient, web‐based	Psychologists, self‐directed	Not identified
Palermo et al.^50^	Cognitive and behavioural skill training for pain management (relaxation techniques, healthy lifestyles, operant strategies for adaptive behaviours), goal setting	Cognitive behavioural theory	Web‐based, family‐based, e‐modules (presented through videos, vignettes, activities, illustrations), e‐mail contact	8 weekly e‐modules	Web‐based	Self‐directed	A generic pre‐existing programme used without adaption for the study population. Intervention development not detailed.
Phillips et al.^51^	SCD education, pain tracking/monitoring, pain management behaviour modification	Paediatric self‐management model	Digital, mobile app	Intervention duration—12 weeks	Web‐based	Self‐guided	CYP with SCD and their parents or caregivers reviewed the intervention and it was revised based on the feedback.
Rodgers‐Melnick et al.^52^	SCD education, pain management, medication management, skill training (relationship‐building), peer support	Not stated	In‐person, group‐based, group‐drumming, songwriting, music production/creation, printed action plans,	4 sessions every 3 months for 12 months	Outpatient	Multidisciplinary team (haematologists, nurse practitioner, social worker, paly patient navigator, music therapist)	Incorporated resources (song stories) developed with CYP with SCD.
Saulsberry et al.^53^	SCD information and transition skills	Not specified	Web‐based, individual‐based, e‐modules, videos, quizzes	6 modules, completed within 1 year	Outpatient	Healthcare professionals (nurse‐led)	Pre‐existing intervention adapted for CYP with SCD. Adaption involved a team of clinicians and hospital administrative staff. No PPI or CYP involvement.
Schatz et al.^54^	Cognitive and behavioural skill training for pain management (deep breathing, relaxation, guided imagery)	Not specified	In‐person, individual‐based, face‐to‐face teaching plus home‐based practices and skill acquisition using a mobile app, written materials, telephone calls	One in‐person session 45–60 min. 8 weeks of engagement with the mobile app	Outpatient, home, telephone	Psychology students. Self‐directed	Modelled from a pre‐existing intervention developed for SCD adult population. No PPI or CYP involvement in the adaption.
Schwartz et al.^55^	Cognitive and behavioural skill training for pain management (deep breathing, relaxation, guided imagery, positive self‐statements)	Not specified	In‐person, individual‐based, face‐to‐face teaching plus home‐based practices using prerecorded audiotapes	4 sessions, sessions 1–3 occur 2 weeks intervals, session 4 a month after 3. Each session is 90 min	Home and telephone‐based	Psychology students and psychologists	Guided imagery content chosen by CYP participants but no PPI or CYP involvement in the development.
Sil et al.^57^	Psychological and behavioural pain management skill training	Dynamic adaption process model	In‐person, group‐based, face‐to‐face teaching (group discussion), videotapes, workbook exercises	3 sessions, 60 min/session over duration of hospitalization	In‐patient	Psychologists	Pre‐existing programme with the educational component adapted for CYP with SCD. Adapted component reviewed by a team of clinicians. No PPI or CYP involvement in the adaption process.
Sil et al.^56^	Cognitive and behavioural education and skill training for pain and illness management (education about pain, behavioural strategies for parents. Relaxation training, activity pacing, cognitive restructuring techniques, healthy lifestyle habits)	Cognitive‐behavioural theory	In‐person, individual‐based, face‐to‐face teaching and skill acquisition	8 bi‐weekly sessions, 45–60 min/session.	Outpatient	Psychologists	Intervention personalized for each CYP‐parent dyad but no PPI or CYP involvement in the development.
Smaldone et al.^58^	SCD and hydroxyurea education, adherence barriers identification, action planning, problem‐solving skills, automated medication text reminders	Not specified	Face‐to‐face teaching, text messages, phone calls, printed brochures	5 sessions, 60–90 min/session	Outpatient, remote	Community health workers	Not specified.
Smith et al.^59^	SCD education, information about transition policy, culture of adult medicine, school and work support and self‐advocacy skill training	Not specified	In‐person, group‐based, individual‐based, face‐to‐face teaching	3 years	Outpatient	Multidisciplinary team (medical providers, educational coordinator, clinical psychologist, social worker, transition coordinator)	Not specified.
Treadwell and Weissman^60^	Medication (deferoxamine) information and management, general information (genetics, nutrition), social support	Not specified	In‐person, group‐based, face‐to‐face teaching, games, visit to a theme park	4 day‐camp	Community	Lay leaders	Not specified
Viola et al.^61^	Educational, skill training and social support mentorship programme focused on transition	Social‐ecological model of adolescent and young adult readiness to transition (SMART)	Web‐based, individual‐based communication via video conferencing and text messaging	Monthly video calls and weekly text messaging for 6 months. Video call last 25–60 min.	Web‐based	Medical students	Content informed by formative need assessment of CYP population.
Wihak et al.^62^	Cognitive and behavioural pain management training (breathing, relaxation, guided imagery), skill training (building confidence and positive thoughts)	Not specified	In‐person, individual‐based, face‐to‐face teaching, audio‐visual resources, workbook	One‐off, 150 min	Outpatient, in‐patient	Multidisciplinary team (haematology nurse, child life provider, social worker)	PPI in the 4‐phased adaptation process of a pre‐existing programme. PPI included clinicians, patient advocate and community consultant. CYP involvement not specified.
Yoon and Godwin^63^	SCD information, self‐care and pain management	Lieberman's theory on interactive health games and health outcomes in children	CD‐ROM, individual‐based, e‐game	3 rounds, one‐off. Intervention duration not indicated	Outpatient	Self‐directed	Pre‐existing programme with the educational component adapted for CYP with SCD. Adapted component reviewed by a team of clinicians and no PPI/CYP involvement.

Abbreviations: CYP, children and young people; SCD, sickle cell disease.

#### 3.3.1 Format and delivery methods

Twelve interventions were delivered both in‐person and remotely,^29^, ^30^, ^32^, ^37^, ^38^, ^39^, ^40^, ^41^, ^42^, ^43^, ^44^, ^47^, ^48^, ^49^, ^54^, ^58^, ^62^ combining lectures, group activities, printed materials, prerecorded audio‐visuals, telephone calls and online features.Combining different delivery methods aimed at promoting flexibility, convenience and financial accessibility, as well as including methods to engage young people. Twelve interventions were technology‐based (e.g., mobile apps, virtual chatrooms, text messaging, e‐modules, electronic or online games, e‐diaries).^33^, ^34^, ^36^, ^44^, ^45^, ^46^, ^48^, ^49^, ^50^, ^51^, ^53^, ^54^, ^58^, ^61^, ^63^, ^64^ Some of these interventions included symptom monitoring and tracking programmes aimed to change and reinforce behaviours believed to promote medical adherence and pain management.

Most interventions were delivered on an individual basis, with only eight interventions (21.1%) being group‐based.^27^, ^28^, ^35^, ^37^, ^52^, ^57^, ^59^, ^60^ Intervention duration ranged from 45 min^38^, ^39^ to 36 months.^59^ Interventions were delivered as one‐off sessions, multiple sessions or were self‐guided. Most were either delivered at a hospital (*n* = 16)^27^, ^28^, ^31^, ^38^, ^39^, ^41^, ^42^, ^43^, ^45^, ^47^, ^52^, ^53^, ^55^, ^56^, ^59^, ^63^ or at multiple locations (*n* = 12) (internet, hospital, telephone or community/home^29^, ^32^, ^36^, ^37^, ^44^, ^48^, ^49^, ^54^, ^57^, ^59^, ^62^, ^64^). One intervention was delivered at a camp.^51^

Most interventions (*n* = 29, 76.3%) were delivered by the researchers themselves or health and social care staff or students, who were predominantly from a psychology background. Only two interventions were delivered by lay counsellors.^35^, ^60^ Some studies explicitly detailed the training and supervision of interventionists and measures to maintain and monitor intervention consistency and fidelity.^30^, ^37^, ^44^, ^47^, ^54^, ^55^, ^58^, ^61^

#### 3.3.2 Intervention development

Several articles (*n* = 13) explicitly stated the frameworks underpinning the interventions' development or implementation. These were predominantly psychosocial and behavioural change theories (Table 8). Only one intervention was underpinned by a self‐management model^48^ and none by the self‐care management model for SCD.^65^ Information on intervention sources or development methods was provided for 27 (84.4%) of the 32 interventions. Thirteen were new interventions developed for the study,^27^, ^29^, ^33^, ^34^, ^36^, ^37^, ^41^, ^46^, ^47^, ^51^, ^52^, ^53^, ^59^, ^64^ six were adapted from existing generic (noncondition specific) self‐management programmes,^33^, ^40^, ^41^, ^48^, ^49^, ^56^ four were self‐management existing programmes for SCD,^42^, ^43^, ^54^, ^63^ and three were generic self‐management interventions implemented without adaptation for SCD.^45^, ^48^, ^50^

Some studies reported involving different lay and professional stakeholders in intervention development or adaptation. However, only five (18.5%) studies involved CYP with SCD in developing their self‐management programmes.^33^, ^40^, ^41^, ^51^, ^61^, ^64^ Of these, one intervention described a co‐creation process where CYP were engaged throughout the development stages.^42^, ^43^, ^54^, ^63^ In the remaining four studies, CYP with SCD appear to have been involved at the initial needs assessment stage^61^ or the review stage following the intervention development.^33^, ^37^, ^51^ CYP involvement appeared limited to intervention content.

In all the studies where race and ethnicity were identified, the SCD population were of African American background. There appeared to be an assumption that participants would be of low socioeconomic status and have strong family systems due to their racial and ethnic identity. These assumptions influenced the intervention format, delivery and target population, with attempts to make interventions financially accessible and/or involve family relatives. Some studies also highlighted the black culture of the SCD population and included black music, sports celebrities, mentors, navigators, interventionists and content from black TV shows to make them ‘culturally sensitive’.^37^, ^47^, ^49^, ^55^, ^61^, ^62^ The agency of CYP, however, appeared to be overlooked, with most interventions developed with minimal or no CYP involvement (see Table 8) to tailor them to the values, expectations and hopes of the CYP. All the interventions were individual‐ or family‐focused, aimed at knowledge, skill acquisition, reinforcement and/or behaviour change, underpinned predominately by psychological models (see Table 8). There was little or no consideration for CYP's wider social contexts like school and/or workplace.

### 3.4 Outcomes measurement

Thirty‐one studies assessed the effectiveness of self‐management interventions using multiple outcome measures (Table 9). Fifteen were feasibility/pilot studies that assessed and reported intervention effectiveness as primary outcomes.^28^, ^35^, ^36^, ^40^, ^44^, ^46^, ^47^, ^48^, ^53^, ^57^, ^58^, ^60^, ^61^, ^63^, ^64^ The most commonly assessed outcomes were disease knowledge, coping, self‐efficacy, quality of life, family and social functioning and support, self‐management confidence and behaviours, healthcare utilization, treatment adherence, parent–child relationship and transition readiness and success. Twenty‐four papers reported on intervention acceptability and feasibility and/or implementation issues. No studies provided data on cost‐effectiveness or intervention development and/or implementation costs.

**Table 9:** Summary of the effectiveness of interventions on outcomes

References	Outcome	Measure	Result or finding (d, day; w, week; m, month)	Study quality rating
Abd Elaziz and Abd Elghany^27^	Knowledge, Pain level, Pain Coping Strategies, Self‐Care Practices	Verbally administered Numerical Rating Scale for pain assessment. Other outcomes were measured using checklists designed for the study.	*3 d*: Improvement in outcomes (*p* < .001)	Moderate
Adegbolagun et al.^28^	Anxiety, Depression, Coping Strategies, Quality of Life (QOL), Knowledge	Hospital Anxiety and Depression Scale, Coping Skills Questionnaire for Sickle Cell Disease‐Adult Version, SF‐36, Knowledge Questionnaire (designed for the study)	*1 w*: Reduction in anxiety (*p* < .001) and depression (*p* < .02). Improvement in active and passive coping skills (*p* < .004), affective coping skills (*p* = .58), self‐reported knowledge, the practice of coping skills and confidence (*p* < .0001)	Moderate
Allemang et al.^29^	Lost to Follow‐up, Appointment Attendance Rate, Medication Adherence, Hospitalization	Medical Records and self‐reported checklists	*12 m*: Reduction in the proportion of transition patients lost to follow‐up (*p* = .00034). Improvement in appointment (*p* = .096), medication adherence (*p* = .047) and hospitalization rates.	Moderate
Barakat et al.^30^	Pain (presence, healthcare use, medication), School Attendance, Health‐Related Hindrance, Coping Strategies, Knowledge, Teen and Family Physical and Psychosocial Well‐being, Family Cohesion	Pain Diary (designed for the study), Health‐related Hindrance Inventory (designed for the study), Coping Strategies Questionnaire, SCD Knowledge Questionnaire, SCD Transition Knowledge Questionnaire (designed for the study), Disease Self‐Efficacy Scale (adapted for this study from a cancer‐specific scale), Child Health Questionnaire	*10 w, 12 m*: No difference in outcomes between groups	Poor
Broome et al.^31^	Coping	School‐agers' Coping Strategies Inventory, Adolescent Coping Orientation for Problem Experiences	*6 w, 12 m* (children): No difference in the frequency of use of coping strategies among the groups. Increase in the number of strategies used and rated effective over time in the intervention groups (*p* < .0003). Nonsignificant reduction in clinic and ER visits and admissions over time. *Last session, 12 m* (adolescents): increase In the frequency of use of coping strategies (*p* = .018). decrease in ER and clinic visits (*p* = .05) and admissions (*p* = .03)	Moderate
Crosby et al.^33^	Clinic Attendance, Hydroxyurea Adherence	Barriers to Care Questionnaire, Web‐based Assessment Tool (Take Charge Programme), Medical Record Review	No real data on the impact of the intervention reported	Moderate
Crosby et al.^35^	General and Disease‐Specific Self‐Efficacy, Self‐Management Behaviours, Health Status, QOL, ER Visits, Patient Activation	SCSES, Self‐Efficacy for Managing Chronic Disease Scale, Transition Readiness Assessment Questionnaire, National Health Interview Survey, Patient Activation Measure, Pediatric QoL Inventory	*6 w, 3 m, 6 m*: significant improvement in general self‐efficacy (*p* = .015) but not in disease‐specific self‐efficacy (*p* = .293), self‐management behaviours (*p* = .39)), health status (*p* = .30), quality of life (*p* = .17) and ER visits (*p* = .59). Medium effect size was noted for patient activation (*p* = .70).	Moderate
Crosby et al.^64^	Health‐Related Quality of Life (HRQOL), Self‐Efficacy, Self‐Management Skills, Knowledge, Health Motivation	Patient Activation Measure, Transition Readiness Questionnaire, Disease‐Specific Knowledge Questionnaire, Treatment Self‐Regulation Questionnaire, Paediatric QoL Inventory SCD Module	*2 w*: Higher scores in quality of life (*p* = .01). Medium effect size in self‐efficacy (*p* = .90). No difference in health motivation, self‐management skills and knowledge (*p* > .05).	Moderate
Cozzi et al.^32^	Self‐Concept, Pain, Anxiety, In‐patient Hospital Visits, Emergency Room (ER) Visits	State‐Trait Anxiety Inventory for adults or children, Tennessee Self‐Concept Scale for adults, Piers‐Harris Children's Self‐Concept Scale, Human Drawings Test	*13 w*: Nonsignificant change in in‐patient and emergency room visits and self‐concept. Improvement in anxiety for children (*p* < .01) and adolescents (*p* < .05); reported headaches as crises symptoms (*p* < .01); reported pain medication days (*p* < .001); reported pain intensity (*p* < .001) and self‐treated crises (*p* < .05).	Moderate
Daniel et al.^37^	HRQOL, School Attendance, Access to School Resources	Haematology/Oncology Psycho‐Educational Needs Assessment, Paediatric QOL Inventory, Woodcock‐Johnson III, Wechsler Abbreviated Scale of Intelligence, Social Problem‐Solving Inventory‐Revised Short Form	*4 w, 6 m*: No impact on all the outcomes.	High
Dobson and Byrne^38^	Self‐Efficacy, School Attendance, Analgesic Use, ER Visits, Pain Intensity	Pain Assessment Tool (investigator‐adapted), Sickle Cell Self‐Efficacy Scale (SCSES), Pain Diary (designed for the study)	*1 m*: improvement in self‐efficacy score (*p* = .000) and pain intensity (*p* = .003), nonsignificant reduction in missed school days, use of analgesics and acute care visits.	Moderate
Dobson^39^	Disease‐specific Self‐Efficacy	SCSES	*1 m*: Greater disease‐specific self‐efficacy (*p* < .000)	Poor
Estepp et al.^40^	Hydroxyurea Adherence, Hospitalization Rates, Clinic Visits	Medication Possession Ratio (days medication in family's possession/days prescribed medication), Haematologic Laboratory Parameters (HbF levels, mean corpuscular volumes, bilirubin levels, absolute reticulocyte counts), Medical Record Review	*3–12 m*: Significant improvement in haematological parameters (*p* < .03) and ER attendance (*p* = .0013). Nonsignificant improvement in hospitalization rates (*p* = .79) and medication possession ratio (*p* = .99). A change in participant's medication possession ratio was the only predictor of improved HbF levels after intervention initiation	Moderate
Fouda et al.^41^	Knowledge, QOL, Self‐Care Practices	Paediatric QOL Inventory. Other outcomes were measured using checklists designed for the study	*3 d, 3 m*: improvement in all outcomes (*p* ≤ .05). Positive correlation between knowledge and QOL and reported self‐care practices and QOL (*p* ≤ .001)	Moderate
Gil et al.^42^	Pain Sensitivity, Coping Strategies	Forgione‐Barber Focal Pressure Stimulator, Coping Strategies Questionnaire for SCD	*1 w*: Lower negative thinking scores (*p* < .05) but nonsignificant interaction effects for coping attempts and illness‐focused strategies. Less pain during low levels of laboratory pain stimulation (*p* < .02). positive and significant correlation between improvement in coping strategies (negative thinking) and improvement in pain sensitivity (*p* < .01)	Poor
Gil et al.^47^	Depression, Anxiety, Pain (sensitivity, sleep quality, functionality, healthcare contacts) Coping Strategies	Children's Depression Inventory, Revised Children's Manifest Anxiety Scale, Pain Diary, Coping Strategies Questionnaire, Structured Pain Interview	*2–3 w, 1 m*: nonsignificant improvement in all outcomes (*p* > .05)	Poor
Green et al.^44^	Hydroxyurea Adherence	Prescription Refill Adherence, Morisky Self‐Report Scale, Foetal Haemoglobin (HbF) level	*1–6 m*: Nonsignificant improvement in pharmacy refill (*p* = .33) and self‐reported medication adherence. Less decrease in Personal best HbF in the intervention group compared to controls (*p* = .009). Controlling for intervention month and group assignment, the intervention group, progressed to Personal best by 2.3% per month during Months 0–4 (*p* = .30).	Moderate
Hazzard et al.^45^	Knowledge, Perceived Social Support, Coping Strategies	How Much Do I Know About SCD Scale, Perceived Social Support‐Friends Scale, Kidcope	*At hospital discharge*: Significant effect on all outcomes (*p* < .001).	Moderate
Hood et al.^46^	Self‐Efficacy, Self‐Management (goal and confidence)	Transition Readiness Assessment Questionnaire, Patient Activation Measure	*6 w*: Nonsignificant improvement in self‐efficacy (*p* > .05). improvement in self‐management confidence, goal‐setting and progress tracking but the significance of improvement is not indicated	Poor
Kaslow et al.^47^	Knowledge, Internalizing and Externalizing Behaviours, Family/Social Functioning and Support	Children's Depression Inventory, Family Adaptability and Cohesion Evaluation Scale, Child Behaviour Checklist, Sickle Cell Disease Knowledge Test (designed for the study)	*6 w*: Improvement in outcomes after the last session (*p* < .01). *6 m*: Improvement in CYP's knowledge sustained. Improvement in parental knowledge and internalizing behaviours reduced (*p* < .05)	High
Ketchen et al.^48^	Knowledge, Health promotion Activities, QOL, Depression, Parent‐Child Relationship Quality	How Much Do I Know About SCD Scale, Health Promotion Activities Checklist (designed for the study), Paediatric QOL Inventory, Children's Depression Inventory, Interaction Behaviour Questionnaire	*At hospital discharge, 2 m*: No significant effect on knowledge, health promotion and depressive symptoms. Significant improvement in QOL (*p* = .05) and parent‐child relationship (*p* = .02))	High
Rodgers‐Melnick et al.^52^	Self‐Efficacy, Trust, Knowledge, ER Visits, Hospitalization	SCSES, Seidman Sickle Cell Knowledge Quiz (designed for the study), Wake Forest Trust in Medical Profession Scale	*3–12 m*: Significant improvement in knowledge (*p* = .0002). No effect on self‐efficacy (*p* = .37), trust (*p* = .08), ER visits (*p* = .33) and hospitalization (*p* = .2)	Poor
Saulsberry et al.^53^	Knowledge, Self‐Management Confidence	Disease Knowledge Tool, Self‐Management Skills Checklist (designed for the study)	*12 m*: Median self‐management confidence score was 8 (range 5–10), and the median knowledge assessment score was 79 (range 37–100). A positive correlation between the number of modules completed and the disease knowledge score (*p* = .003). No correlation was found between the number of modules completed and the self‐management confidence ratings (*p* = .945).	Moderate
Schatz et al.^54^	Coping Strategies, Pain (frequency, intensity, functionality)	Coping Strategies Questionnaire for SCD, Daily Pain and Activity Diary (electronic)	*2 m*: Increase in self‐reported use of cognitive‐behavioural skills (*p* = .03) and beliefs in pain controllability (*p* = .02).	Moderate
Sil et al.^57^	Knowledge, Pain Coping Efficacy	Knowledge of Psychological Interventions for Pain, Child Pain Coping Self‐Efficacy Scale	*End of each session*: Only significant improvement postintervention for session 1 (<.01). No significant difference in coping scores and self‐efficacy remained relatively similar across all sessions	Moderate
Sil et al.^56^	Healthcare Utilization, Treatment Adherence, Pain Intensity, Functional Disability, Pain Coping Efficacy	Medical Record Review, Numeric Rating Scale (designed for the study), Functional Disability Inventory, Pain Coping Questionnaire	*Every 2–3 sessions, 8 w*: No significant improvement in healthcare utilization (*p* > .17). 31.6% of participants in the treatment group reported a statistically significant decrease in pain intensity, functional disability and improved coping efficacy.	Poor
Smaldone et al.^58^	Hydroxyurea Adherence, HRQOL, Youth‐Parent Self‐Management Responsibility Concordance	Paediatric QOL Inventory, Paediatric QOL SCD Module, Sickle Cell Family Responsibility Scale (adapted from the Diabetes Family Responsibility Questionnaire)	*3 m*: Improved dyad self‐management responsibility concordance (3.5 points, 95% CI: −0.2, 7.1). *6 m*: Improved generic HRQOL (9.8 points, 95% CI: 0.4, 19.2), No difference in QOL	Moderate
Smith et al.^59^	Transition Readiness, Disease‐Related Self‐Efficacy, Disease‐Related Stress, Feelings about Transition, Transfer Success, Patient Retention	Transition Intervention Programme‐Readiness For Transition Assessment Tool (designed for the study), SCSES, Feelings‐Sickle Cell Transfer Questionnaire, Sickle Cell Stress Scale‐Adolescent (designed for the study)	*36 m*: Improvements in all outcomes but statistical/clinical significance not indicated	Poor
Treadwell and Weissman^60^	Treatment Compliance, Knowledge, Perceived Social Support, Parent‐Child Responsibility for Medication Management	24‐h recall interview, Multi‐Choice Questionnaires (designed for the study), Perceived Competence Scale for Children	*2 w*: Nonsignificant improvement in outcomes	Moderate
Viola et al.^61^	Transition Readiness, Disease‐Related QOL, Disease‐Related Self‐Efficacy, Health Literacy, Knowledge	Sickle Cell Transition Intervention Programme‐Readiness for Transition, Adult Sickle Cell QOL Measurement System, SCSES, Morisky Medication Adherence Scale	*6 m*: Significant improvement in transition readiness (*p* < .01) and self‐efficacy (*p* = .002). Nonsignificant improvement in QOL, health literacy and knowledge	High
Yoon and Godwin^63^	Knowledge, Self‐Management Confidence	Knowledge checklist (designed for the study), 100 mm horizontal Visual Analogy Scale	*0 d*: improvement in knowledge (*p* = .010) and self‐management confidence (*p* = .001)	Moderate

Abbreviations: CYP, children and young people; HRQOL, health‐related quality of life; QOL, quality of life; SCD, sickle cell disease.

Outcomes were measured at different time points between the final intervention session and 12 months postintervention implementation (Table 9). Only four studies assessed outcomes over a longer period.^30^, ^31^, ^52^, ^60^ Three studies measured several CYP‐focused outcomes via proxy reports.^27^, ^51^, ^58^ These studies also employed measurement tools not validated for CYP. Further, several studies involving self‐guided interventions did not assess engagement levels, and only two^48^, ^53^ accounted for the connection between engagement and outcomes. No studies reported if CYP with SCD participated in deciding how self‐management interventions' effectiveness, acceptability and feasibility should be evaluated.

Different validated and unvalidated generic and SCD‐specific outcome measurement tools were used (Table 4). Most tools had not been formally evaluated for use with CYP with SCD, and different tools were used to measure the same outcomes across the papers (Table 9). Only three papers^44^, ^58^, ^60^ reported the measurement of physiological parameters (Table 9). Most of the studies reporting intervention effectiveness failed to report on the effect size and/or 95% confidence intervals of the results. The evidence below must, therefore, be considered in the context of these limitations. Table 9 summarizes the effectiveness data.

### 3.5 Effectiveness

Seventeen studies (20 articles) evaluated the impact of self‐management programmes on psycho‐behavioural outcomes (self‐efficacy, anxiety, depression and coping).^28^, ^30^, ^31^, ^32^, ^34^, ^36^, ^38^, ^39^, ^42^, ^43^, ^45^, ^46^, ^47^, ^48^, ^52^, ^54^, ^57^, ^58^, ^59^, ^61^ Findings from five studies associated self‐management interventions with statistically significant positive impacts on self‐efficacy.^28^, ^38^, ^39^, ^61^, ^64^ In one study, improved self‐efficacy was correlated with improved self‐management confidence and behaviours.^46^, ^64^ Similarly, five studies reported significant improvements in coping and the use of positive coping strategies.^27^, ^28^, ^45^, ^54^, ^56^ Coping outcomes were reported primarily for pain. Mixed findings were reported regarding self‐management interventions' impact on pain frequency and intensity.^30^, ^37^, ^42^, ^43^, ^49^, ^54^, ^56^ Only one study reported significant improvement in anxiety and depression postintervention.^28^ The effective interventions for psycho‐behavioural outcomes were predominantly in‐person, hospital‐based and multisessional.

The overall strength of the evidence base for the psycho‐behavioural outcomes limits confidence in the results, particularly since 16 of the 20 articles were of moderate to poor methodological quality. Only one high‐quality study associated the self‐management intervention (e‐mentorship by medical students) with significant improvements in self‐efficacy.^61^ Furthermore, effectiveness appeared to reduce overtime for self‐efficacy and coping.^30^, ^31^, ^47^, ^52^

Eight studies of mixed quality examined the impact of self‐management interventions on healthcare utilization which included emergency room visits, in‐patient admission duration and/or hospitalization rate.^29^, ^30^, ^32^, ^35^, ^38^, ^40^, ^52^, ^56^ Only a 12‐month CYP‐personalized transition programme delivered by a designated social worker reported a statistically significant reduction in hospitalization rate at 12 months follow‐up.^29^ This study involved the largest number of participants (*n* = 110); however, outcome measurement was based on a retrospective record review, which would not have included hospitalizations outside the study centre.

Health‐related quality of life was measured in eight studies.^28^, ^35^, ^36^, ^37^, ^41^, ^48^, ^58^, ^61^ Only three studies of moderate quality reported statistically significant improvement in quality of life.^28^, ^41^, ^64^ However, in two, improvement was only observed in the social domain.^28^, ^64^ The high‐quality studies identified no significant improvement in quality of life.^37^, ^61^

Overall, our greatest confidence is in the evidence of improvements in SCD knowledge and social outcomes, where studies of predominantly moderate methodological quality reported positive outcomes (Table 9). Sixteen studies of strong to moderate quality reported a statistically significant impact of self‐management interventions on these outcomes.^27^, ^29^, ^36^, ^38^, ^40^, ^41^, ^44^, ^45^, ^47^, ^48^, ^52^, ^57^, ^58^, ^59^, ^60^, ^61^, ^63^ Eight studies found that educational programmes on SCD and pain improved knowledge and understanding.^27^, ^40^, ^47^, ^52^, ^57^, ^60^, ^63^, ^64^ Six studies found a positive association between improved knowledge and understanding and self‐management confidence, behaviours and practices.^27^, ^41^, ^46^, ^53^, ^63^, ^64^ Similarly, six studies also found improvements in social functioning and perceived social support.^38^, ^45^, ^47^, ^48^, ^59^, ^60^ Although evidence on knowledge and social outcomes is based upon high‐quality studies, many used different unvalidated measurement tools and had small sample sizes and short‐term follow‐ups.

Six studies measured medical adherence as medication adherence, CYP responsibility for treatment, clinic attendance or transition success.^29^, ^40^, ^44^, ^58^, ^60^, ^61^ Four studies reported significant improvements in these outcomes.^29^, ^44^, ^58^ The effective interventions were behavioural and skills training programmes delivered by a designated social care provider^29^, ^44^, ^58^ and an educational programme delivered by lay counsellors.^60^ None of the technological interventions using treatment reminders improved treatment adherence.^40^, ^61^

### 3.6 Acceptability and engagement

Ten RCTs,^30^, ^37^, ^43^, ^44^, ^46^, ^47^, ^49^, ^50^, ^55^, ^58^ eight quasi‐experimental studies,^28^, ^32^, ^45^, ^51^, ^52^, ^57^, ^61^, ^62^ three cross‐sectional studies,^33^, ^35^ two cohort studies^40^, ^53^ and one qualitative study^64^ reported on participants' satisfaction and engagement with the interventions. Levels of satisfaction and engagement were measured by survey and were assessed only for participants who completed the interventions. Studies reported high levels of satisfaction and acceptance, with those associated with the highest satisfaction levels being interventions that were delivered in person or remotely;^28^, ^30^, ^32^, ^33^, ^34^, ^35^, ^36^, ^37^, ^44^, ^47^, ^51^, ^55^, ^61^, ^62^, ^64^ involved peers with SCD or parents^32^, ^34^, ^45^, ^46^, ^47^, ^52^ and supported pain coping skills and strategies,^30^, ^32^, ^34^, ^46^, ^47^ SCD knowledge and understanding,^30^, ^34^, ^46^, ^47^, ^52^ advocacy skills^46^ and direct interaction with care providers.^40^, ^61^ Participants valued interventions that were engaging, interactive, motivating and enjoyable.^33^, ^34^, ^35^, ^36^, ^37^, ^46^, ^52^, ^64^

However, participants' engagement with digital interventions was reported to be variable, decreasing over time. Engagement with interventions was reported to be higher amongst parent participants than CYP for the interventions that involved CYP‐parent dyads.^44^, ^51^, ^58^ In addition, engagement levels were reported to be higher among younger CYP participants than in the older CYP cohort in the same studies.^34^, ^43^, ^46^, ^47^

Some studies reported on CYP participants' feedback regarding the negative aspects of the interventions and their preferences.^44^, ^47^, ^50^, ^58^, ^61^, ^64^ Negative aspects included the burden of home‐based self‐directed activities,^47^ the lack of mobile phone‐based technology interventions,^50^, ^64^ the frequency of reminder notifications and the surveillance features of some interventions.^44^, ^50^, ^58^, ^61^ Interestingly, participants recommended incorporating in‐person sessions for interventions involving virtual platforms for social interaction and strengthening peer support.^61^, ^64^ Some studies described how the professionals delivering the interventions^31^, ^61^ reported improved communication and relationships with CYP and improved understanding of their challenges.

### 3.7 Evidence related to self‐management intervention implementation

The main challenges reported for intervention implementation related to study recruitment and retention and provider (interventionist) availability. Eleven studies reported high follow‐up attrition rates, which ranged between 32%^40^ and 53%^57^ (Table 2). Participants' responses for not completing the intervention and/or outcome assessments included the burden of completing outcome measures,^44^ loss of interest,^37^, ^52^, ^54^ dislike of intervention format,^50^, ^54^ acute illness and hospitalization,^35^, ^48^, ^57^ and transport and time constraints.^48^, ^56^ Some eligible participants were excluded or lost to follow‐up due to the intervention times being limited to the clinic's working hours. Barriers to participation and engagement in the m‐Health interventions included device and technical malfunctions.^46^, ^49^, ^54^ Implementation facilitators included patient navigation systems, study schedule flexibility, parental, family and clinical staff involvement, third‐sector organization partnership and accessible m‐Health programmes.^31^, ^34^, ^37^, ^46^, ^48^, ^51^, ^52^ The only healthcare system implementation issue reported was provider availability^57^, ^59^ when competing for clinical demands and emergency consultations often interfered with intervention delivery. Training each multidisciplinary team member to deliver self‐management interventions was reportedly facilitative. Several studies made recommendations to facilitate future intervention acceptability and engagement (Table 10).

**Table 10:** Recommendations for future self‐management interventions

Self‐management programmes/interventions need to:	m‐Health self‐management interventions need to:
Be personalized to CYP's goals and values.^34^, ^45^, ^46^ Be family‐oriented.^30^ Use multiple formats and delivery approaches to enhance flexibility, convenience, acceptability, usefulness and engagement.^37^, ^50^ Include components to facilitate communication with health and social care professionals.^50^ Develop the broad range of knowledge and skills for managing SCD.^50^ Support older CYP's capacity to navigate psychological, relational, educational, vocational, economic and health and social care system challenges.^29^, ^30^, ^43^, ^47^, ^64^ Be integrated within the wider healthcare system.^46^ Involve schools.^47^ Designate a lead for self‐management in the multidisciplinary team to support CYP.^30^, ^59^, ^61^, ^62^	Employ user‐centred design.^51^ Mitigate risks to privacy.^46^ Consider time, resources, monitoring and technology maintenance.^46^ Include features to allow CYP to self‐assess their progress and intervention impact.^51^ Help CYP communicate health, illness and self‐management information with health care professionals by enabling them to capture visual feedback in real‐time to share.^51^

Abbreviations: CYP, children and young people; SCD, sickle cell disease.

## 4 DISCUSSION

The development and evaluation of self‐management interventions to support CYP with SCD is a growing area of research, particularly in the United States, with 50% of the included papers being published in the last 5 years. This paper reports the first comprehensive review of the evidence on their effectiveness and acceptability. In terms of their characteristics, the interventions developed are wide‐ranging in relation to their purpose, content, format and delivery methods. However, only a third of these (10/32) demonstrated statistically significant improvements in the outcomes measured.^27^, ^28^, ^29^, ^38^, ^39^, ^41^, ^45^, ^47^, ^54^, ^63^ These interventions were delivered in‐person, involved educational components, skills training for pain management and medical adherence and/or promoted family and/or peer support; and reported improvements in knowledge, social functioning and/or medical adherence. However, it is unclear if these improvements are maintained over time. Similar to the findings of previous reviews on CYP with chronic conditions,^66^, ^67^ the majority of the included interventions were focused on medical management (i.e., disease‐specific and healthcare‐related) and/or emotional management (i.e., feelings and intrinsic characteristics). This notwithstanding, no clear effects were found in favour of any interventions aimed at symptom reduction.

Only a third of the technological interventions (4/12) showed effectiveness. These findings are consistent with the findings of other reviews in which limited data was found to support the effectiveness of mobile or web‐based applications for self‐management of adolescents with a chronic illness^68^ and for treatment adherence among CYP and adults with SCD.^19^, ^21^ There was limited evidence for interventions improving healthcare utilization or quality of life. Conflicting and inconsistent evidence for the effectiveness of self‐management interventions on quality of life in CYP with chronic illnesses has been found and reported by others.^66^ In addition to previous research on the effectiveness of self‐management for CYP with chronic illnesses,^66^ this review revealed that Interventions that combined technological platforms and in‐person group‐based approaches and involved family and care providers appeared to be more acceptable to CYP.

However, confidence in these results is limited by the methodological quality of the included papers. Most interventions are in the early stages of evaluation, and most papers report findings from retrospective observational studies, single‐arm pre‐ and poststudies or small feasibility/pilot trials. Several studies were characterized by high follow‐up attrition rates and demonstrated significant potential risk for bias. There were inconsistencies and wide variations in the outcome measurements used across the studies, with some unvalidated tools. This reflects the current state of limited availability of validated health‐related outcome tools for CYP with SCD specifically and the SCD population in general. Further, none of the studies compared the different types of intervention components or examined if specific self‐management intervention components are more or less effective and for which patient and health and social setting characteristics, which would allow for creating targeted interventions.

Consequently, more robust definitive RCTs conducted across multiple and different settings are needed. Future trials should be methodologically robust in terms of randomization, larger sample size and power calculations, blinding and outcome measures (i.e., use of age‐appropriate validated tools, effect size calculations, clinical significance indications and longer follow‐up periods). Qualitative studies should be nested within future trials to help understand intervention effectiveness and acceptability. This is particularly important as patient‐reported outcomes measures (e.g., quality of life, psychosocial well‐being) are highly variable and qualitative methods will provide additional explanatory information that can facilitate the interpretation of trial outcomes. More economic evaluations of interventions are also needed. In addition, more research is needed to develop and validate health‐related outcome measurement tools for CYP with SCD to support intervention and programme evaluations.

The lack of, or where sought, the minimal involvement of CYP in the intervention development and implementation and study design and conduct is a limitation of the included studies. This may explain why several interventions included features that CYP found burdensome and unhelpful, as well as problems with recruitment, retention and engagement. A recent review suggests that involving CYP in designing and delivering self‐management interventions improves effectiveness.^69^ Ignoring the agency of CYP, particularly from minority backgrounds like CYP with SCD, also has the potential to create and deepen health disparities and inequalities. This supports embedded co‐production to ensure self‐management intervention design and implementation are consistent with CYP's needs, voices, experiences and expectations rather than prioritizing medical adherence.

Further, interventions focused on role management (i.e., social domains and social participation) were limited. Despite the importance of the social context on self‐management,^6^, ^7^, ^70^, ^71^ the included studies overlooked home, school and/or work environments. This is despite several research studies reporting the difficulties of engaging in SCD self‐management practices in these settings.^9^, ^11^, ^13^, ^14^, ^15^ The lack of accommodation for SCD self‐management practices in the home, school and workplace has been highlighted, including how school policies and practices contradict self‐management.^9^, ^11^, ^13^, ^14^, ^15^ At the same time, self‐management practices may be (dis)regarded as interference and perceived as disruptive and stigmatizing by others and CYP themselves.^9^, ^10^, ^11^, ^13^, ^14^, ^15^, ^72^ Future interventions should, therefore, consider how families and school and/or work contexts can support CYP's ability to self‐management.

Including CYP with SCD from diverse backgrounds or developing interventions inclusive of diverse cultures is important. However, no study included CYP with SCD from Eastern Mediterranean, Middle Eastern and Southeast Asian backgrounds. Indeed, SCD appeared to be ‘problematised’ as a ‘black illness’ in the included studies. Future research should, therefore, strive to involve CYP with SCD from non‐Black backgrounds. This could be achieved through cross‐country, cross‐institutional and cross‐centre research collaborations; development of interventions that are diverse (in terms of language and content); targeted recruitment of non‐Black populations and more non‐US‐based studies.

Overall, the highlighted limitations mean that there is limited evidence for any single self‐management programme or programme components in relation to promoting health and social outcomes for CYP with SCD. However, we found effective and acceptable interventions appear to include those that provide support from peers and trusted adults (e.g., parents, teachers, relatives and care providers). While the ultimate goal for self‐management is to increase CYP with SCD autonomy and independence in managing their illness, they depend on others for encouragement, communication and treatment support. Adults (e.g., parents, relatives, care providers, teachers and adults with SCD) can provide information on how CYP can independently handle health and social care needs, while peers can learn from and support one another by sharing personal experiences. A recent review highlights the pivotal role peers and adults play in supporting the adoption and promotion of self‐management strategies in CYP with chronic conditions.^69^ Future interventions should, therefore, include components that facilitate peer and significant adult support. The involvement of peers, families, care providers and teachers is particularly important because CYP with SCD have been found to experience significant stigma in relation to their illness management, even from families, care providers and teachers due to their illness and race/ethnicity.^9^, ^11^, ^72^, ^73^

## 5 STRENGTHS AND LIMITATIONS

This is the first review of self‐management interventions for CYP with SCD that synthesizes the evidence from the range of programmes developed, the range of outcomes assessed and the diversity of evaluative study designs. Including young people with SCD in the review's conduct has enhanced the credibility of the process. However, there are some limitations. Only peer‐reviewed articles published in English were included, posing a potential publication bias. It was impossible to synthesize study results using meta‐analysis due to the heterogeneity of the interventions and outcome measures. As previously described, the robustness of the included studies limits the confidence that can be placed on the results. In addition, the cost‐effectiveness or cost‐benefits of interventions has not been evaluated, and there is a lack of qualitative research to inform understanding of intervention effectiveness and acceptability.

## 6 CONCLUSION

Findings generated from this systematic review provide a critical summary of the characteristics, effectiveness, acceptability and feasibility of self‐management interventions for CYP with SCD. This review found that interventions that promoted peer, parent/family and care provider support and combined technology and in‐person group methods appear to be associated with the effectiveness and acceptability to CYP. However, there is limited evidence for any single self‐management programme or programme components in relation to promoting health/social outcomes for CYP with SCD. Future studies need to be robust in design, conducted across multiple and different settings and collect long‐term outcome data to assess the effectiveness, acceptability, cost‐benefits and generalizability of interventions across populations and settings. In addition, it is important that more validated health‐related outcome tools are developed for CYP with SCD; CYP are involved in intervention development, study design and implementation; CYP participants from non‐Black backgrounds are included and that attention is paid to the wider context of CYP's lives when designing and evaluating self‐management interventions.

## CONFLICT OF INTEREST

The authors declare no conflict of interest.

## Supporting information

Supporting information.

Supporting information.

## Data Availability

Data sharing does not apply to this article as no data sets were generated or analysed during the current study.

## References

[1] Piel FB , Hay SI , Gupta S , Weatherall DJ , Williams TN . Global burden of sickle cell anaemia in children under five, 2010‐2050: modelling based on demographics, excess mortality, and interventions. PLoS Med. 2013;10:e1001484. 23874164 10.1371/journal.pmed.1001484 PMC3712914

[2] Preiss DJ . The young child with sickle cell disease. Adv Nurse Pract. 1998;6(6):32‐39. 9708052

[3] Rees DC , Williams TN , Gladwin MT . Sickle‐cell disease. The Lancet. 2010;376(9757):2018‐2031. 10.1016/S0140-6736(10)61029-X 21131035

[4] Platt OS , Brambilla DJ , Rosse WF , et al. Mortality in sickle cell disease – life expectancy and risk factors for early death. N Engl J Med. 1994;330(23):1639‐1644. 7993409 10.1056/NEJM199406093302303

[5] Powars DR , Chan LS , Hiti A , Ramicone E , Johnson C . Outcome of sickle cell anemia: a 4‐decade observational study of 1056 patients. Medicine. 2005;84(6):363‐376. 16267411 10.1097/01.md.0000189089.45003.52

[6] Lorig KR , Holman HR . Self‐management education: history, definition, outcomes and mechanisms. Ann Behav Med. 2003;26(1):1‐7. 12867348 10.1207/S15324796ABM2601_01

[7] Schulman‐Green D , Jaser S , Martin F , et al. Processes of self‐management in chronic illness. J Nurs Scholarsh. 2012;44(2):136‐144. 22551013 10.1111/j.1547-5069.2012.01444.x PMC3366425

[8] Nightingale R , McHugh G , Kirk S , Swallow V . Supporting children and young people to assume responsibility from their parents for the self‐management of their long‐term condition: An integrative review. Child Care Health Dev. 2019;45(2):175‐188. 30690751 10.1111/cch.12645

[9] Poku BA , Caress A‐L , Kirk S . “Body as a machine”: how adolescents with sickle cell disease construct their fatigue experiences. Qual Health Res. 2020;30:1431‐1444. 32406309 10.1177/1049732320916464

[10] Poku BA , Caress A‐L , Kirk S . Adolescents' experiences of living with sickle cell disease: An integrative narrative review of the literature. Int J Nurs Stud. 2018;80:20‐28. 29353708 10.1016/j.ijnurstu.2017.12.008

[11] Poku BA , Pilnick A . Biographical accounts of the impact of fatigue in young people with sickle cell disease. Sociol Health Illn. 2022;44(6):1027‐1046. 35488415 10.1111/1467-9566.13477 PMC9545386

[12] Dyson SM , Abuateya H , Atkin K , et al. Reported school experiences of young people living with sickle cell disorder in England. Br Educ Res J. 2010;36(1):125‐142.

[13] Dyson SM , Atkin K , Culley LA , Dyson SE , Evans H . Sickle cell, habitual dys‐positions and fragile dispositions: young people with sickle cell at school. Sociol Health Illn. 2011;33(3):465‐483. 21375541 10.1111/j.1467-9566.2010.01301.x PMC3084516

[14] Dyson SM , Atkin KM , Berghs MJ , Greene AM . On the possibility of a disabled life in capitalist ruins: black workers with sickle cell disorder in England. Soc Sci Med. 2021;272:113713. 33540149 10.1016/j.socscimed.2021.113713

[15] Berghs M , Dyson S , Gabba A , et al. I want to become someone!” gender, reproduction and the moral career of motherhood for women with sickle cell disorders. Cult Health Sex. 2022:1‐15. 10.1080/13691058.2022.2083239 35678290

[16] Modi AC , Pai AL , Hommel KA , et al. Pediatric self‐management: a framework for research, practice and policy. Pediatrics. 2012;129(2):e473‐e485. 22218838 10.1542/peds.2011-1635 PMC9923567

[17] Paterson GA , Nayda RJ , Paterson JA . Chronic condition self‐management: working in partnership toward appropriate models for age and culturally diverse clients. Contemp Nurse. 2012;40(2):169‐178. 22554211 10.5172/conu.2012.40.2.169

[18] Edwards LY , Edwards CL . Psychosocial treatments in pain management of sickle cell disease. J Natl Med Assoc. 2010;102(11):1084‐1094. 21141299 10.1016/s0027-9684(15)30737-9

[19] Shih S , Cohen LL . A systematic review of medication adherence interventions in pediatric sickle cell disease. J Pediatr Psychol. 2020;45(6):593‐606. 32417887 10.1093/jpepsy/jsaa031

[20] Viola A , Porter J , Shipman J , Brooks E , Valrie C . A scoping review of transition interventions for young adults with sickle cell disease. Pediatr Blood Cancer. 2021;68:e29135. 34089222 10.1002/pbc.29135 PMC8316342

[21] Badawy SM , Cronin RM , Hankins J , et al. Patient‐centered eHealth interventions for children, adolescents and adults with sickle cell disease: systematic review. J Med Internet Res. 2018;20(7):e10940. 30026178 10.2196/10940 PMC6072976

[22] Stern C , Lizarondo L , Carrier J , et al. Methodological guidance for the conduct of mixed methods systematic reviews. JBI Evidence Synthesis. 2020;18(10):2108‐2118. 32813460 10.11124/JBISRIR-D-19-00169

[23] Page MJ , McKenzie JE , Bossuyt PM , et al. The PRISMA 2020 statement: an updated guideline for reporting systematic reviews. Syst Rev. 2021;10(89):89. 10.1186/s13643-021-01626-4 33781348 PMC8008539

[24] Joanne Briggs Institute System for the Unified Management, Assessment and Review of Information (JBI SUMARI). Accessed November 24, 2021. https://sumari.jbi.global/ 10.11124/JBISRIR-2016-002421 27846109

[25] Kirk S , Beatty S , Callery P , Milnes L , Pryjmachuk S . Evaluating self‐care support for children and young people with long‐term conditions. National Institute for Health and Social Care. 2010. Accessed May 30, 2022. http://www.netscc.ac.uk/hsdr/projdetails.php?ref=08-1715-162

[26] Pryjmachuk S , Elvey R , Kirk S , Kendal S , Bower P , Catchpole R . Developing a model of mental health self‐care support for children and young people through an integrated evaluation of available types of provision involving systematic review, meta‐analysis and case study. Health Serv Del Res. 2014;2(18):1‐212. 25642499

[27] Abd Elaziz SM , Abd , Elghany RM . Effect of self care management program on pain and fatigue in sickle cell children. Int J Novel Res Healthc Nurs. 2019;6(3):747‐761.

[28] Adegbolagun A , Ani C , Adejumo O , James B , Omigbodun O . Effect of a group‐based cognitive behavioural therapy program on the psychological wellbeing, quality of life and coping of students with sickle cell disease in Nigeria. Int J Disabil Dev Educ. 2020;69:1095‐1104. 10.1080/1034912X.2020.1755422

[29] Allemang B , Allan K , Johnson C , et al. Impact of a transition program with navigator on loss to follow‐up, medication adherence, and appointment attendance in hemoglobinopathies. Pediatr Blood Cancer. 2019;66:e27781. 31045326 10.1002/pbc.27781

[30] Barakat LP , Schwartz LA , Salamon KS , Radcliffe J . A family‐based randomized controlled trial of pain intervention for adolescents with sickle cell disease. J Pediatr Hematol Oncol. 2010;32(7):540‐547. 20686425 10.1097/MPH.0b013e3181e793f9 PMC2950888

[31] Broome ME , Maikler V , Kelber S , Bailey P , Lea G . An intervention to increase coping and reduce health care utilization for school‐age children and adolescents with sickle cell disease. J Natl Black Nurses Assoc. 2001;12(2):6‐14. 11902023

[32] Cozzi L , Tryon WW , Sedlacek K . The effectiveness of biofeedback‐assisted relaxation in modifying sickle cell crises. Biofeedback Self‐Regul. 1987;12(1):51‐61. 3663738 10.1007/BF01000078

[33] Crosby LE , Barach I , McGrady ME , Kalinyak KA , Eastin AR , Mitchell MJ . Integrating interactive web‐based technology to assess adherence and clinical outcomes in pediatric sickle cell disease. Anemia. 2012;2012:1‐8. 10.1155/2012/492428 PMC3372407 22701785

[34] Crosby LE , Ware RE , Goldstein A , et al. Development and evaluation of iManage: a self‐management app co‐designed by adolescents with sickle cell disease. Pediatr Blood Cancer. 2017;64(1):139‐145. 27574031 10.1002/pbc.26177 PMC7354646

[35] Crosby LE , Joffe NE , Peugh J , Ware RE , Britto MT . Pilot of the chronic disease self‐management program for adolescents and young adults with sickle cell disease. J Adolesc Health. 2017;60(1):120‐123. 27793727 10.1016/j.jadohealth.2016.08.022 PMC5182081

[36] Crosby LE , Hood A , Kidwell K , et al. Improving self‐management in adolescents with sickle cell disease. Pediatr Blood Cancer. 2020;67(10):e.28492. 10.1002/pbc.28492 PMC7722105 32697889

[37] Daniel LC , Li Y , Smith K , et al. Lessons learned from a randomized controlled trial of a family‐based intervention to promote school functioning for school‐age children with sickle cell disease. J Pediatr Psychol. 2015;40(10):1085‐1094. 26136404 10.1093/jpepsy/jsv063 PMC4626743

[38] Dobson CE , Byrne MW . Original research: using guided imagery to manage pain in young children with sickle cell disease. Am J Nurs. 2014;114(4):26‐36. 10.1097/01.NAJ.0000445680.06812.6a 24632887

[39] Dobson C . Outcome results of self‐efficacy in children with sickle disease pain who were trained to use guided imagery. Appl Nurs Res. 2015;28:384‐390. 26608443 10.1016/j.apnr.2014.12.005

[40] Estepp JH , Winter B , Johnson M , Smeltzer MP , Howard SC , Hankins JS . Improved hydroxyurea effect with the use of text messaging in children with sickle cell anemia. Pediatr Blood Cancer. 2014;61:2031‐2036. 25132074 10.1002/pbc.25177

[41] Mahdy Fouda N , Mohamed Adly R , Shafik Mahmoud F , Abd el‐Ghany mohamed R . Effect of self‐learning guidelines on quality of life and self‐care reported practice of adolescents with sickle cell anemia. J Nurs Sci Benha Univ. 2021;2:400‐415.

[42] Gil KM , Wilson JJ , Edens JL , et al. Cognitive coping skills training in children with sickle cell disease pain. Int J Behav Med. 1997;4(4):364‐377. 16250724 10.1207/s15327558ijbm0404_7

[43] Gil KM . Daily coping practice predicts treatment effects in children with sickle cell disease. J Pediatr Psychol. 2001;26(3):163‐173. 11259518 10.1093/jpepsy/26.3.163

[44] Green NS , Manwani D , Matos S , et al. Randomized feasibility trial to improve hydroxyurea adherence in youth ages 10‐18 years through community health workers: the HABIT study. Pediatr Blood Cancer. 2017;64(12):e26689. 10.1002/pbc.26689 PMC6538388 28643377

[45] Hazzard A , Celano M , Collins M , Markov Y . Effects of STARBRIGHT world on knowledge, social support, and coping in hospitalized children with sickle cell disease and asthma. Child Health Care. 2002;31(1):69‐86.

[46] Hood AM , Nwankwo C , Walton A , et al. Mobile health use predicts self‐efficacy and self‐management in adolescents with sickle cell disease. Transl Behav Med. 2021;11:1823‐1831. 33949674 10.1093/tbm/ibab041 PMC8541694

[47] Kaslow NJ , Collins MH , Rashid FL , et al. The efficacy of a pilot family psychoeducational intervention for pediatric sickle cell disease (SCD). Fam Syst Health. 2000;18(4):381‐404.

[48] Ketchen B , Hazzard A , Lassiter S , et al. STARBRIGHT world: a pilot study of a home‐based sickle cell psychoeducational intervention. Chil Health Care. 2006;35(4):321‐338.

[49] McClellan CB , Schatz JC , Puffer E , Sanchez CE , Stancil MT , Roberts CW . Use of handheld wireless technology for a home‐based sickle cell pain management protocol. J Pediatr Psychol. 2009;34(5):564‐573. 19029141 10.1093/jpepsy/jsn121

[50] Palermo TM , Dudeney J , Santanelli JP , Carletti A , Zempsky WT . Feasibility and acceptability of internet‐delivered cognitive behavioral therapy for chronic pain in adolescents with sickle cell disease and their parents. J Pediatr Hematol Oncol. 2018;40(2):122‐127. 29176462 10.1097/MPH.0000000000001018 PMC5820201

[51] Levin ME , Petersen JM , Durward C , et al. A randomized controlled trial of online acceptance and commitment therapy to improve diet and physical activity among adults who are overweight/obese. Transl Behav Med. 2021;11:1216‐1225. 10.1093/tbm/ibaa123 33289785

[52] Rodgers‐Melnick SN , Pell TJG , Lane D , et al. The effects of music therapy on transition outcomes in adolescents and young adults with sickle cell disease. Int J Adolesc Med Health. 2019;31:e20170004. 10.1515/ijamh-2017-0004 28779565

[53] Saulsberry AC , Hodges JR , Cole A , Porter JS , Hankins J . Web‐based technology to improve disease knowledge among adolescents with sickle cell disease: pilot study. JMIR Pediatr Parent. 2020;3(1):e15093. 10.2196/15093 31909718 PMC6996770

[54] Schatz J , Schlenz AM , McClellan CB , et al. Changes in coping, pain, and activity after cognitive‐behavioral training: a randomized clinical trial for pediatric sickle cell disease using smartphones. Clin J Pain. 2015;31(6):536‐547. 25503599 10.1097/AJP.0000000000000183 PMC4424076

[55] Schwartz LA , Radcliffe J , Barakat LP . The development of a culturally sensitive pediatric pain management intervention for African American adolescents with sickle cell disease. Chil Health Care. 2007;36(3):267‐283. 10.1080/02739610701377954 PMC2733520 19718272

[56] Sil S , Lai K , Lee JL , et al. Preliminary evaluation of the clinical implementation of cognitive‐behavioral therapy for chronic pain management in pediatric sickle cell disease. Complement Ther Med. 2020;49:102348. 10.1016/j.ctim.2020.102348 32147059 PMC7092728

[57] Sil S , Lee JL , Klosky J , et al. The comfort ability program for adolescents with sickle cell pain: Evaluating feasibility and acceptability of an inpatient group‐based clinical implementation. Pediatr Blood Cancer. 2021;68(6):e29013. 10.1002/pbc.29013 33742546 PMC8085908

[58] Smaldone A , Findley S , Manwani D , Jia H , Green NS . HABIT, a randomized feasibility trial to increase hydroxyurea adherence, suggests improved health‐related quality of life in youths with sickle cell disease. J Pediatr. 2018;197:177‐185. 29571930 10.1016/j.jpeds.2018.01.054 PMC5970970

[59] Smith WR , Sisler IY , Johnson S , et al. Lessons learned from building a pediatric‐to‐adult sickle cell transition program. South Med J. 2019;112(3):190‐197. 30830235 10.14423/SMJ.0000000000000950 PMC6590675

[60] Treadwell MJ , Weissman L . Improving adherence with deferoxamine regimens for patients receiving chronic transfusion therapy. Sem Hematol. 2001;38(suppl 1):77‐84. 10.1016/s0037-1963(01)90064-2 11206966

[61] Viola AS , Drachtman R , Kaveney A , et al. Feasibility of medical student mentors to improve transition in sickle cell disease. J Pediatr Psychol. 2021;46(6):650‐661. 33779756 10.1093/jpepsy/jsab031 PMC8291672

[62] Wihak T , Burns M , Miranda J , Windmueller G , Oakley C , Coakley R . Development and feasibility testing of the comfort ability program for sickle cell pain: a patient‐informed, video‐based pain management intervention for adolescents with sickle cell disease. Clin Pract Pediatr Psychol. 2020;8(2):150‐163.

[63] Yoon SL , Godwin A . Enhancing self‐management in children with sickle cell disease through playing a CD‐ROM educational game: a pilot study. Pediatr Nurs. 2007;33(1):60‐63. 17411004

[64] Crosby LE , Joffe NE , Kidwell KM , et al. Perceptions of a self‐management intervention for adolescents with sickle cell disease. Clin Pract Pediatr Psychol. 2022;10(1):79‐90.

[65] Jenerette CM , Murdaugh C . Testing the theory of self‐care management for sickle cell disease. Res Nurs Health. 2008;31(4):355‐369. 18247376 10.1002/nur.20261

[66] Bal MI , Sattoe JNT , Roelofs PDDM , Bal R , van Staa A , Miedema HS . Exploring effectiveness and effective components of self‐management interventions for young people with chronic physical conditions: A systematic review. Patient Educ Couns. 2016;99(8):1293‐1309. 26954345 10.1016/j.pec.2016.02.012

[67] Sattoe JNT , Bal MI , Roelofs PDDM , Bal R , Miedema HS , van Staa A . Self‐management interventions for young people with chronic conditions: a systematic overview. Patient Educ Couns. 2015;98(6):704‐715. 25819373 10.1016/j.pec.2015.03.004

[68] Virella Pérez YI , Medlow S , Ho J , Steinbeck K . Mobile and web‐based apps that support self‐management and transition in young people with chronic illness: systematic review. J Med Internet Res. 2019;21(11):e13579. 10.2196/13579 31746773 PMC6893564

[69] Camp‐Spivey LJ , Logan A , Nichols M . Theoretical and contextual considerations for self‐management strategies of children and adolescents with chronic diseases: an integrative review. J Child Health Care. 2021;26(0):242‐261. 10.1177/13674935211013697 33913767

[70] Ong BN , Rogers A , Kennedy A , et al. Behaviour change and social blinkers? The role of sociology in trials of self‐management behaviour in chronic conditions. Sociol Health Illn. 2014;36(2):226‐238. 24528304 10.1111/1467-9566.12113

[71] Renedo A , Miles S , Marston C . Transitions to adulthood: self‐governance and disciplining in the making of patient citizens. Sociol Health Illn. 2020;42(3):481‐495. 31663619 10.1111/1467-9566.13019 PMC7078962

[72] Poku BA , Pilnick A , Kirk S . How a child's gender mediates maternal care and expectations in the fatigue experiences of adolescents with sickle cell disease. J Fam Stud. 2022:1‐22. 10.1080/13229400.2022.2060851

[73] Poku BA , Pilnick A. Research knowledge transfer to improve care and support for adolescents with sickle cell disease in Ghana. Health Expect. 2022. 10.1111/hex.13573 PMC9615053 35909322

