# Supplementary material for: Self‐management interventions for children and young people with sickle cell disease: A systematic review
Source: Health Expect. 2023 Jan 3;26(2):579–612. doi: 10.1111/hex.13692 (PMC10010100; doi:10.1111/hex.13692)
Supplement: Supplementary file 1 — Supporting information. [file HEX-26--s001.docx]

**Supplementary File 1: The search strategies used across the 8 databases**

**Database: Ovid MEDLINE(R)**

**Date searched: 10 November 2021**

**Date range searched: 1946 to 9 November 2021**

**Record retrieved: 169**

**Search Strategy:**

| **#** | **Searches** | **Results** |
| --- | --- | --- |
| 1 | Self Administration/ | 11907 |
| 2 | self administer*.ti,ab. | 40592 |
| 3 | (self administer* adj2 (questionnaire* or survey* or interview*)).ti,ab. | 26062 |
| 4 | 2 not 3 | 14530 |
| 5 | Self Care/ | 34791 |
| 6 | (self care* or selfcaring or selfcare* or self-care* or self caring).ti,ab. | 20545 |
| 7 | (self manag* or selfmanag* or self-manag*).ti,ab. | 22866 |
| 8 | (selfassess* or self assess* or self-assess*).ti,ab. | 17405 |
| 9 | (self monitor* or selfmonitor* or self-monitor*).ti,ab. | 8830 |
| 10 | (selfdiagnos* or self diagnos* or self-diagnos*).ti,ab. | 854 |
| 11 | (selfreport* or self report* or self-report*).ti,ab. | 177205 |
| 12 | (selfpolic* or self polic* or self-polic* or selfsupervis* or self supervis* or self-supervis*).ti,ab. | 383 |
| 13 | (selfhlep* or self help* or self-help*).ti,ab. | 6939 |
| 14 | (selftreat* or self treat* or self-treat*).ti,ab. | 1833 |
| 15 | (selfmedicat* or self medicat* or self-medicat* or selfcure* or self cure* or self-cure* or selfcuring or self curing or self-curing).ti,ab. | 6106 |
| 16 | (selfrecover* or self recover* or self-recover*).ti,ab. | 432 |
| 17 | (self initia* or self-initiat* or selfregulat* or self regulat* or self-regulat*).ti,ab. | 15006 |
| 18 | 1 or 4 or 5 or 6 or 7 or 8 or 9 or 10 or 11 or 12 or 13 or 14 or 15 or 16 or 17 | 304910 |
| 19 | Social Support/ | 75474 |
| 20 | social support*.ti,ab. | 44783 |
| 21 | (peer adj (support or advice or advis* or monitor* or intervention* or train* or instruct* or consult* or assist* or educat* or information or mentor*)).ti,ab. | 8398 |
| 22 | (group adj (support or advice or advis* or monitor* or intervention* or train* or instruct* or consult* or assist* or educat* or information)).ti,ab. | 8740 |
| 23 | ((mutual or telephone or online or virtual) adj support).ti,ab. | 2270 |
| 24 | (expert patient* or virtual communit* or online communit*).ti,ab. | 1704 |
| 25 | (befriend* or coach* or mentor* or buddy or buddies).ti,ab. | 36250 |
| 26 | health trainer*.ti,ab. | 79 |
| 27 | Behavior Therapy/ | 29241 |
| 28 | ((behaviour* or behavior*) adj (manag* or modif* or therap*)).ti,ab. | 31835 |
| 29 | Psychotherapy/ | 55844 |
| 30 | Psychotherapy, Brief/ | 3634 |
| 31 | Psychotherapy, Group/ | 14315 |
| 32 | psychoeducat*.ti,ab. | 5854 |
| 33 | Cognitive Behavioral Therapy/ | 28010 |
| 34 | cognitive behavioural therapy.ti,ab. | 4329 |
| 35 | motivational therap*.ti,ab. | 44 |
| 36 | (skill training or coping skill* or empower*).ti,ab. | 35053 |
| 37 | Adaptation, Psychological/ | 99757 |
| 38 | adaptive behav*.ti,ab. | 5175 |
| 39 | Mindfulness/ | 4709 |
| 40 | Meditation/ | 3269 |
| 41 | Problem Solving/ | 25824 |
| 42 | exp Communication/ | 335862 |
| 43 | interpersonal communication.ti,ab. | 1645 |
| 44 | recovery.ti,ab. | 486426 |
| 45 | (CBT or cognitive therap* or congnitive behav*).ti,ab. | 15153 |
| 46 | Motivational Interviewing/ | 2254 |
| 47 | Patient Education as Topic/ | 87587 |
| 48 | patient education.ti,ab. | 19195 |
| 49 | ((patient or consumer health) adj (education or information)).ti,ab. | 27815 |
| 50 | (patient adj2 (educat* or advice or advis* or instruct* or train* or coach*)).ti,ab. | 28168 |
| 51 | (nurse adj educator*).ti,ab. | 3836 |
| 52 | (teaching adj (material* or pamplet* or resource*)).ti,ab. | 1479 |
| 53 | (educational adj (material* or leaflet* or booklet* or toolkit*)).ti,ab. | 5080 |
| 54 | Bibliotherapy/ | 425 |
| 55 | Patient Participation/ | 27939 |
| 56 | patient participation.ti,ab. | 2449 |
| 57 | consumer participation.ti,ab. | 316 |
| 58 | (involv* or participat* or collaborat* or empower* or enabl* or engag* or partner*).ti,ab. | 3796373 |
| 59 | (decision* adj2 (shared or support* or aid or adis or making)).ti,ab. | 184004 |
| 60 | Decision Making/ | 100752 |
| 61 | exp Diet/ | 304732 |
| 62 | exp Exercise/ | 220863 |
| 63 | Telemedicine/ | 31101 |
| 64 | (telemedicine or telecare or telenursing or telemonitor* or telehealth or ehealth).ti,ab. | 25180 |
| 65 | Remote Consultation/ | 5385 |
| 66 | ((telephone or remote or phone) adj2 (follow* or support* or consult* or advice or advis* or intervention* or instruct* or assist* or educat* or information or monitor*)).ti,ab. | 16426 |
| 67 | Cell Phone/ | 9364 |
| 68 | Telephone/ | 12669 |
| 69 | (cell phone* or cellphone* or telephone* or cellular phone* or cellular telephone* or mobile telephone* or mobile phone*).ti,ab. | 76253 |
| 70 | (handheld computer* or hand held computer* or smartphone* or smart phone*).ti,ab. | 17399 |
| 71 | (microcomputer* or micro computer* or tablet* or ipad).ti,ab. | 64540 |
| 72 | (iphone or android* or blackberry).ti,ab. | 4680 |
| 73 | (wireless communication* or mobile communication* or wireless technolog* or mobile techonolog*).ti,ab. | 3262 |
| 74 | (mobile app* moblie application* or software or portable software app* or portable mobile application* or pda or pdas or mobile game* or personal digital assistan*).ti,ab. | 208381 |
| 75 | Text Messaging/ | 3839 |
| 76 | Reminder Systems/ | 3698 |
| 77 | (text messag* or texting or text* or short message service or SMS).ti,ab. | 182889 |
| 78 | Electronic Mail/ | 2848 |
| 79 | (email* or e-mail* or e-mailing).ti,ab. | 19410 |
| 80 | (ehealth or e-health or mhealth or m-health or mcare).ti,ab. | 9835 |
| 81 | (remote consult* or teleconsult*).ti,ab. | 2041 |
| 82 | (internet or online or virtual or remote or telephon*).ti,ab. | 405208 |
| 83 | Blogging/ | 1055 |
| 84 | blog*.ti,ab. | 2090 |
| 85 | Social Media/ | 11844 |
| 86 | (website* or website*).ti,ab. | 32187 |
| 87 | (chat room* or chatroom* or webchat* or web chat* or social network* or social forum*).ti,ab. | 20420 |
| 88 | or/19-87 | 6034497 |
| 89 | exp Child/ | 2024812 |
| 90 | Adolescent/ | 2137415 |
| 91 | (schoolchild* or school child* or teen* or teenager* or young person or young people or young adult* or emerging adult* or youth* or adolescen* cild or children or student* or juvenile* or youngster* or kid or kids or pediatric or paediatric).ti,ab. | 1814906 |
| 92 | 89 or 90 or 91 | 3896108 |
| 93 | Anemia, Sickle Cell/ | 22317 |
| 94 | sickle cell.ti,ab. | 26000 |
| 95 | (sickle cell disease* or sickle cell syndrome or sickle cell disorder* or sickle cell anaemia or sickle cell anemia).ti,ab. | 22224 |
| 96 | (SCD or SCA or HbSS or HBSS or HBSC or HbSC).ti,ab. | 23094 |
| 97 | (haemoglobin S or hemoglobin S or haemoglobin SC or hemoglobin SC).ti,ab. | 1882 |
| 98 | 93 or 94 or 95 or 96 or 97 | 46193 |
| 99 | 18 and 88 and 92 and 98 | 169 |

**Database: Ovid Embase**

**Date searched: 11 November 2021**

**Date range searched: 1974 to 10 November 2021**

**Record retrieved: 452**

**Search Strategy:**

| **#** | **Searches** | **Results** |
| --- | --- | --- |
| 1 | self administer*.ti,ab. | 52857 |
| 2 | (self administer* adj2 (questionnaire* or survey* or interview*)).ti,ab. | 32622 |
| 3 | 1 not 2 | 20235 |
| 4 | self care/ | 65813 |
| 5 | (self care* or selfcaring or selfcare* or self-care* or self caring).ti,ab. | 29394 |
| 6 | (self manag* or selfmanag* or self-manag*).ti,ab. | 33371 |
| 7 | (selfassess* or self assess* or self-assess* or selfdiagnos* or self diagnos* or self-diagnos*).ti,ab. | 26284 |
| 8 | (selfreport* or self report* or self-report*).ti,ab. | 234972 |
| 9 | (selfmonitor* or self monitor* or self monitor* or self policing or selfpolicing or self-policing or selfsupervis* or self supervis* or self-supervis*).ti,ab. | 12955 |
| 10 | (selfhelp* or self help* or self-help*).ti,ab. | 8883 |
| 11 | (selftreat* or self treat* or self-treat* or selfmedicat* or self medicat* or self-medicat* or selfcure* or self cure* or self-cure* or selfcuring or self curing or self-curing).ti,ab. | 11251 |
| 12 | (selfrecover* or self recover* or self-recover*).ti,ab. | 446 |
| 13 | (self initiat* or self-initiat* or selfregulat* or self regulat* or self-regulat*).ti,ab. | 17399 |
| 14 | drug self administration/ | 12162 |
| 15 | 3 or 4 or 5 or 6 or 7 or 8 or 9 or 10 or 11 or 12 or 13 or 14 | 406562 |
| 16 | social support/ | 100778 |
| 17 | social support*.ti,ab. | 55651 |
| 18 | (group adj (support or advice or advis* or monitor* or intervention* or train* or instruct* or consult* or assist* or educat* or information)).ti,ab. | 13662 |
| 19 | (peer adj (support or advice or advis* or monitor* or intervention* or train* or instruct* or consult* or assist* or educat* or information or mentor*)).ti,ab. | 11447 |
| 20 | ((mutual or telephone or online or virtual) adj support).ti,ab. | 3218 |
| 21 | (expert patient* or virtual communit* or online communit*).ti,ab. | 2398 |
| 22 | (befriend* or coach* or mentor* or buddy or buddies).ti,ab. | 47042 |
| 23 | health trainer*.ti,ab. | 105 |
| 24 | exp psychotherapy/ | 266654 |
| 25 | group therapy/ | 19660 |
| 26 | psychoeducat*.ti,ab. | 8574 |
| 27 | cognitive therapy/ | 43719 |
| 28 | cognitive behavioural therapy.ti,ab. | 6113 |
| 29 | cognitive behavorial therapy.ti,ab. | 2 |
| 30 | motivational therap*.ti,ab. | 72 |
| 31 | motivational interviewing/ | 5754 |
| 32 | (skill training or coping skill* or empower*).ti,ab. | 47369 |
| 33 | psychological adjustment/ | 1543 |
| 34 | psychological adaptation.ti,ab. | 801 |
| 35 | adaptive behav*.ti,ab. | 6345 |
| 36 | exp behavior therapy/ | 63412 |
| 37 | behaviour therapy.ti,ab. | 3590 |
| 38 | ((behaviour* or behavior*) adj (manag* or modif* or therap*)).ti,ab. | 43929 |
| 39 | mindfulness/ | 10842 |
| 40 | meditation/ | 8126 |
| 41 | problem solving/ | 36443 |
| 42 | exp interpersonal communication/ | 690064 |
| 43 | recovery.ti,ab. | 640053 |
| 44 | (CBT or cognitive therap* or cognitive behav*).ti,ab. | 52057 |
| 45 | patient education/ | 118449 |
| 46 | ((patient or consumer health) adj (education or information)).ti,ab. | 43636 |
| 47 | (patient adj2 (educat* or advice or advis* or instruct* or train* or coach*)).ti,ab. | 44867 |
| 48 | (nurse adj educator*).ti,ab. | 4224 |
| 49 | (teaching adj (material* or pamplet* or resource*)).ti,ab. | 2249 |
| 50 | (educational adj (material* or leaflet* or booklet* or toolkit*)).ti,ab. | 8033 |
| 51 | bibliotherapy/ | 277 |
| 52 | patient participation/ | 30550 |
| 53 | patient participation.ti,ab. | 3316 |
| 54 | consumer participation.ti,ab. | 363 |
| 55 | (involv* or participat* or collaborat* or empower* or enabl* or engag* or partner*).ti,ab. | 4809664 |
| 56 | (decision* adj2 (shared or support* or aid or adis or making)).ti,ab. | 250720 |
| 57 | decision making/ | 248152 |
| 58 | exp diet/ | 353465 |
| 59 | exp exercise/ | 375139 |
| 60 | telemedicine/ | 34057 |
| 61 | (telemedicine or telecare or telenursing or telemonitor* or telehealth or ehealth).ti,ab. | 33162 |
| 62 | teleconsultation/ | 12375 |
| 63 | remote consultation.ti,ab. | 365 |
| 64 | ((telephone or remote or phone) adj2 (follow* or support* or consult* or advice or advis* or intervention* or instruct* or assist* or educat* or information or monitor*)).ti,ab. | 28842 |
| 65 | mobile phone/ | 19177 |
| 66 | telephone/ | 40829 |
| 67 | (cell phone* or cellphone* or telephone* or cellular phone* or cellular telephone* or mobile telephone* or mobile phone*).ti,ab. | 105732 |
| 68 | (handheld computer* or hand held computer* or smartphone* or smart phone*).ti,ab. | 23367 |
| 69 | (microcomputer* or micro computer* or micro-computer* or tablet* or ipad).ti,ab. | 104442 |
| 70 | (iphone or android* or blackberry).ti,ab. | 7218 |
| 71 | (wireless communication* or mobile communication* or wireless technolog* or mobile techonolog*).ti,ab. | 3529 |
| 72 | (mobile app* or moblie application* or software or portable software app* or portable mobile application* or pda or pdas or mobile game* or personal digital assistan*).ti,ab. | 344356 |
| 73 | text messaging/ | 6167 |
| 74 | reminder system/ | 2846 |
| 75 | reminder system*.ti,ab. | 1088 |
| 76 | (text messag* or texting or text* or short message service or SMS).ti,ab. | 197056 |
| 77 | e-mail/ | 25337 |
| 78 | electronic mail.ti,ab. | 918 |
| 79 | (email* or e-mail* or e-mailing).ti,ab. | 38621 |
| 80 | (ehealth or e-health or mhealth or m-health or mHealth or mCare or mcare).ti,ab. | 11290 |
| 81 | (internet or online or virtual or remote or telephon*).ti,ab. | 542674 |
| 82 | blogging/ | 501 |
| 83 | blog*.ti,ab. | 3252 |
| 84 | social media/ | 30976 |
| 85 | (website* or website*).ti,ab. | 47710 |
| 86 | (chat room* or chatroom* or webchat* or web chat* or social network* or social forum*).ti,ab. | 23981 |
| 87 | or/16-86 | 7947697 |
| 88 | exp child/ | 2804759 |
| 89 | exp adolescent/ | 1626232 |
| 90 | exp juvenile/ | 3677209 |
| 91 | (schoolchild* or school child* or teen* or teenager* or young person or young people or young adult* or emerging adult* or youth* or adolescen* child or children or student* or juvenile* or youngster* or kid or kids or pediatric or paediatric).ti,ab. | 2335331 |
| 92 | 88 or 89 or 90 or 91 | 4495710 |
| 93 | exp sickle cell/ | 3203 |
| 94 | (sickle cell anaemia or sickle cell anemia).ti,ab. | 9777 |
| 95 | (sickle cell disease* or sickle cell disorder* or sickle cell syndrome).ti,ab. | 25465 |
| 96 | (SCD or SCA or HbSS or HBSS or HBSC or HbSC).ti,ab. | 41282 |
| 97 | (haemoglobin S or hemoglobin S or haemoglobin SC or hemoglobin SC).ti,ab. | 2542 |
| 98 | 93 or 94 or 95 or 96 or 97 | 61494 |
| 99 | 15 and 87 and 92 and 98 | 452 |

**Database: APA PsycInfo (via OVIDSP)**

**Date searched: 11 November 2021**

**Date range searched: 1806 to November Week 2 2021**

**Record retrieved: 71**

**Search Strategy:**

| **#** | **Searches** | **Results** |
| --- | --- | --- |
| 1 | exp Drug Self Administration/ | 2488 |
| 2 | self administer*.ti,ab. | 14511 |
| 3 | (self administer* adj2 (questionnaire* or survey* or interview*)).ti,ab. | 7727 |
| 4 | 2 not 3 | 6784 |
| 5 | exp Self-Care/ | 3008 |
| 6 | exp Self-Care Skills/ | 10915 |
| 7 | (self care* or selfcaring or selfcare* or self-care* or self caring).ti,ab. | 10618 |
| 8 | (self manag* or selfmanag* or self-manag*).ti,ab. | 10907 |
| 9 | (selfassess* or self assess* or self-assess* or selfdiagnos* or self diagnos* or self-diagnos*).ti,ab. | 9322 |
| 10 | (selfreport* or self report* or self-report*).ti,ab. | 134907 |
| 11 | (selfmonitor* or self monitor* or self monitor* or self policing or selfpolicing or self-policing or selfsupervis* or self supervis* or self-supervis*).ti,ab. | 6591 |
| 12 | (selfhelp* or self help* or self-help*).ti,ab. | 8677 |
| 13 | (selftreat* or self treat* or self-treat* or selfmedicat* or self medicat* or self-medicat* or selfcure* or self cure* or self-cure* or selfcuring or self curing or self-curing).ti,ab. | 2336 |
| 14 | (selfrecover* or self recover* or self-recover*).ti,ab. | 52 |
| 15 | (self initiat* or self-initiat* or selfregulat* or self regulat* or self-regulat*).ti,ab. | 24265 |
| 16 | 1 or 4 or 5 or 6 or 7 or 9 or 10 or 11 or 12 or 13 or 14 or 15 | 206823 |
| 17 | exp Social Support/ | 39298 |
| 18 | social support.ti,ab. | 50021 |
| 19 | (group adj (support or advice or advis* or monitor* or intervention* or train* or instruct* or consult* or assist* or educat* or information)).ti,ab. | 8814 |
| 20 | (peer adj (support or advice or advis* or monitor* or intervention* or train* or instruct* or consult* or assist* or educat* or information or mentor*)).ti,ab. | 7431 |
| 21 | ((mutual or telephone or online or virtual) adj support).ti,ab. | 2030 |
| 22 | (expert patient* or virtual communit* or online communit*).ti,ab. | 2852 |
| 23 | (befriend* or coach* or mentor* or buddy or buddies).ti,ab. | 38231 |
| 24 | health trainer*.ti,ab. | 28 |
| 25 | exp Motivational Interviewing/ | 2722 |
| 26 | exp Cognitive Therapy/ | 13720 |
| 27 | exp Psychotherapy/ | 210539 |
| 28 | exp Group Psychotherapy/ | 23437 |
| 29 | cognitive behavioural therapy.ti,ab. | 3790 |
| 30 | cognitive behavioral therapy.ti,ab. | 13934 |
| 31 | motivational therapy.ti,ab. | 28 |
| 32 | (skill training or coping skill* or empower*).ti,ab. | 37923 |
| 33 | exp Emotional Adjustment/ | 22308 |
| 34 | psychological adaptation.ti,ab. | 855 |
| 35 | psychological adjustment.ti,ab. | 5256 |
| 36 | exp Adaptive Behavior/ | 3641 |
| 37 | adaptive behav*.ti,ab. | 6385 |
| 38 | exp Behavior Therapy/ | 21770 |
| 39 | behaviour therapy.ti,ab. | 2804 |
| 40 | ((behaviour* or behavior*) adj (manag* or modif* or therap*)).ti,ab. | 42984 |
| 41 | exp Mindfulness/ | 11006 |
| 42 | exp Mindfulness-Based Interventions/ | 1784 |
| 43 | exp Meditation/ | 5085 |
| 44 | exp Problem Solving/ | 49638 |
| 45 | exp Communication/ | 327740 |
| 46 | interpersonal communication.ti,ab. | 2882 |
| 47 | (CBT or cognitive therap* or cognitive behav*).ti,ab. | 50865 |
| 48 | recovery.ti,ab. | 70851 |
| 49 | exp Client Education/ | 4280 |
| 50 | patient education.ti,ab. | 3326 |
| 51 | ((patient or client or consumer health) adj (education or information)).ti,ab. | 4758 |
| 52 | (patient adj2 (educat* or advice or advis* or instruct* or train* or coach*)).ti,ab. | 5231 |
| 53 | (nurse adj educator*).ti,ab. | 1156 |
| 54 | instructional material*.ti,ab. | 1574 |
| 55 | (teaching adj (material* or pamplet* or resource*)).ti,ab. | 1288 |
| 56 | ((educational or instructional) adj2 (material* or leaflet* or booklet* or toolkit*)).ti,ab. | 3622 |
| 57 | exp Bibliotherapy/ | 781 |
| 58 | exp Client Participation/ | 2587 |
| 59 | patient participation.ti,ab. | 880 |
| 60 | (involv* or participat* or collaborat* or empower* or enabl* or engag* or partner*).ti,ab. | 1105219 |
| 61 | (decision* adj2 (shared or support* or aid or adis or making)).ti,ab. | 102087 |
| 62 | exp Decision Making/ | 130088 |
| 63 | exp Exercise/ | 28788 |
| 64 | exp Diets/ | 18309 |
| 65 | exp Telemedicine/ | 10301 |
| 66 | (telemedicine or teleconsultation or remote consultation or telecare or telenursing or telemonitor* or telehealth or ehealth).ti,ab. | 4745 |
| 67 | exp Mobile Phones/ | 6228 |
| 68 | exp Telephone Systems/ | 8373 |
| 69 | (cell phone* or cellphone* or telephone* or cellular phone* or cellular telephone* or mobile telephone* or mobile phone*).ti,ab. | 30737 |
| 70 | (handheld computer* or hand held computer* or smartphone* or smart phone*).ti,ab. | 5990 |
| 71 | (microcomputer* or micro computer* or micro-computer* or tablet* or ipad).ti,ab. | 8012 |
| 72 | (wireless communication* or mobile communication* or wireless technolog* or mobile techonolog*).ti,ab. | 696 |
| 73 | (mobile app* or moblie application* or software or portable software app* or portable mobile application* or pda or pdas or mobile game* or personal digital assistan*).ti,ab. | 29545 |
| 74 | exp Text Messaging/ | 1216 |
| 75 | reminder system.ti,ab. | 79 |
| 76 | (text messag* or texting or text* or short message service or SMS).ti,ab. | 112751 |
| 77 | (email* or e-mail* or e-mailing or electronic mail*).ti,ab. | 10524 |
| 78 | (ehealth or e-health or mhealth or m-health or mHealth or mCare or mcare).ti,ab. | 2423 |
| 79 | (internet or online or virtual or remote or telephon*).ti,ab. | 180478 |
| 80 | exp Websites/ or exp Blog/ or exp Social Media/ or exp Internet/ or exp Electronic Communication/ or exp Computer Mediated Communication/ | 61171 |
| 81 | blogging.ti,ab. | 745 |
| 82 | (website* or web site*).ti,ab. | 17754 |
| 83 | (chat room* or chatroom* or webchat* or web chat* or social network* or social forum*).ti,ab. | 27702 |
| 84 | (iphone or android* or blackberry).ti,ab. | 875 |
| 85 | or/17-84 | 2041329 |
| 86 | (schoolchild* or school child* or teen* or teenager* or young person or young people or young adult* or emerging adult* or youth* or adolescen* child or children or student* or juvenile* or youngster* or kid or kids or pediatric or paediatric).ti,ab. | 1169609 |
| 87 | exp Sickle Cell Disease/ | 1140 |
| 88 | sickle cell.ti,ab. | 1543 |
| 89 | (sickle cell anaemia or sickle cell anemia).ti,ab. | 261 |
| 90 | (sickle cell disease* or sickle cell disorder* or sickle cell syndrome).ti,ab. | 1267 |
| 91 | (SCD or SCA or HbSS or HBSS or HBSC or HbSC).ti,ab. | 2599 |
| 92 | (haemoglobin S or hemoglobin S or haemoglobin SC or hemoglobin SC).ti,ab. | 13 |
| 93 | 87 or 88 or 89 or 90 or 91 or 92 | 3350 |
| 94 | 16 and 85 and 86 and 93 | 71 |

**Database: CINAHL (EBSCO)**

**Date searched: 11 November 2021**

**Date range searched: 1987 to 4 November 2021**

**Record retrieved: 179**

**Search Strategy:**

| S97 | S16 AND S85 AND S89 AND S96 | Expanders - Apply equivalent subjects  Search modes - Boolean/Phrase | Interface - EBSCOhost Research Databases  Search Screen - Advanced Search  Database - CINAHL Plus | 179 |
| --- | --- | --- | --- | --- |
| S96 | S90 OR S91 OR S92 OR S93 OR S94 OR S95 | Expanders - Apply equivalent subjects  Search modes - Boolean/Phrase | Interface - EBSCOhost Research Databases  Search Screen - Advanced Search  Database - CINAHL Plus | 10,761 |
| S95 | TI (“haemoglobin S” or “hemoglobin S” or “haemoglobin SC” or “hemoglobin SC”) OR AB (“haemoglobin S” or “hemoglobin S” or “haemoglobin SC” or “hemoglobin SC”) | Expanders - Apply equivalent subjects  Search modes - Boolean/Phrase | Interface - EBSCOhost Research Databases  Search Screen - Advanced Search  Database - CINAHL Plus | 192 |
| S94 | TI (SCD or SCA or HbSS or HBSS or HBSC or HbSC) OR AB (SCD or SCA or HbSS or HBSS or HBSC or HbSC) | Expanders - Apply equivalent subjects  Search modes - Boolean/Phrase | Interface - EBSCOhost Research Databases  Search Screen - Advanced Search  Database - CINAHL Plus | 5,500 |
| S93 | TI (“sickle cell disease*” or “sickle cell disorder*” or “sickle cell syndrome”) OR AB (“sickle cell disease*” or “sickle cell disorder*” or “sickle cell syndrome”) | Expanders - Apply equivalent subjects  Search modes - Boolean/Phrase | Interface - EBSCOhost Research Databases  Search Screen - Advanced Search  Database - CINAHL Plus | 4,529 |
| S92 | TI (“sickle cell anaemia” or “sickle cell anemia”) OR AB (“sickle cell anaemia” or “sickle cell anemia”) | Expanders - Apply equivalent subjects  Search modes - Boolean/Phrase | Interface - EBSCOhost Research Databases  Search Screen - Advanced Search  Database - CINAHL Plus | 1,168 |
| S91 | TI "sickle cell" OR AB "sickle cell" | Expanders - Apply equivalent subjects  Search modes - Boolean/Phrase | Interface - EBSCOhost Research Databases  Search Screen - Advanced Search  Database - CINAHL Plus | 6,315 |
| S90 | (MH "Anemia, Sickle Cell+") | Expanders - Apply equivalent subjects  Search modes - Boolean/Phrase | Interface - EBSCOhost Research Databases  Search Screen - Advanced Search  Database - CINAHL Plus | 5,905 |
| S89 | S86 OR S87 OR S88 | Expanders - Apply equivalent subjects  Search modes - Boolean/Phrase | Interface - EBSCOhost Research Databases  Search Screen - Advanced Search  Database - CINAHL Plus | 1,390,908 |
| S88 | TI (schoolchild* or “school child*” or teen* or teenager* or “young person” or “young people” or “young adult*” or “emerging adult*” or youth* or adolescen* child or children or student* or juvenile* or youngster* or kid or kids or pediatric or paediatric) OR AB (schoolchild* or “school child*” or teen* or teenager* or “young person” or “young people” or “young adult*” or “emerging adult*” or youth* or adolescen* child or children or student* or juvenile* or youngster* or kid or kids or pediatric or paediatric) | Expanders - Apply equivalent subjects  Search modes - Boolean/Phrase | Interface - EBSCOhost Research Databases  Search Screen - Advanced Search  Database - CINAHL Plus | 823,525 |
| S87 | (MH "Adolescence+") | Expanders - Apply equivalent subjects  Search modes - Boolean/Phrase | Interface - EBSCOhost Research Databases  Search Screen - Advanced Search  Database - CINAHL Plus | 566,251 |
| S86 | (MH "Child+") | Expanders - Apply equivalent subjects  Search modes - Boolean/Phrase | Interface - EBSCOhost Research Databases  Search Screen - Advanced Search  Database - CINAHL Plus | 716,846 |
| S85 | S17 OR S18 OR S19 OR S20 OR S21 OR S22 OR S23 OR S24 OR S25 OR S26 OR S27 OR S28 OR S29 OR S30 OR S31 OR S32 OR S33 OR S34 OR S35 OR S36 OR S37 OR S38 OR S39 OR S40 OR S41 OR S42 OR S43 OR S44 OR S45 OR S46 OR S47 OR S48 OR S49 OR S50 OR S51 OR S52 OR S53 OR S54 OR S55 OR S56 OR S57 OR S58 OR S59 OR S60 OR S61 OR S62 OR S63 OR S64 OR S65 OR S66 OR S67 OR S68 OR S69 OR S70 OR S71 OR S72 OR S73 OR S74 OR S75 OR S76 OR S77 OR S78 OR S79 OR S80 OR S81 OR S82 OR S83 OR S84 | Expanders - Apply equivalent subjects  Search modes - Boolean/Phrase | Interface - EBSCOhost Research Databases  Search Screen - Advanced Search  Database - CINAHL Plus | 2,077,774 |
| S84 | TI (telemedicine or teleconsultation or "remote consultation" or telecare or telenursing or telemonitor* or telehealth or ehealth) OR AB (telemedicine or teleconsultation or "remote consultation" or telecare or telenursing or telemonitor* or telehealth or ehealth) | Expanders - Apply equivalent subjects  Search modes - Boolean/Phrase | Interface - EBSCOhost Research Databases  Search Screen - Advanced Search  Database - CINAHL Plus | 15,916 |
| S83 | (MH "Telemedicine+") OR (MH "Telehealth+") | Expanders - Apply equivalent subjects  Search modes - Boolean/Phrase | Interface - EBSCOhost Research Databases  Search Screen - Advanced Search  Database - CINAHL Plus | 29,745 |
| S82 | TI ("electronic communication" or "computer mediated communication") OR AB ("electronic communication" or computer mediated communication") | Expanders - Apply equivalent subjects  Search modes - Boolean/Phrase | Interface - EBSCOhost Research Databases  Search Screen - Advanced Search  Database - CINAHL Plus | 716 |
| S81 | TI blogging OR AB blogging | Expanders - Apply equivalent subjects  Search modes - Boolean/Phrase | Interface - EBSCOhost Research Databases  Search Screen - Advanced Search  Database - CINAHL Plus | 384 |
| S80 | TI (iphone or android* or blackberry) OR AB (iphone or android* or blackberry) | Expanders - Apply equivalent subjects  Search modes - Boolean/Phrase | Interface - EBSCOhost Research Databases  Search Screen - Advanced Search  Database - CINAHL Plus | 2,052 |
| S79 | TI (website* or “web site*”) OR AB (website* or “web site*”) | Expanders - Apply equivalent subjects  Search modes - Boolean/Phrase | Interface - EBSCOhost Research Databases  Search Screen - Advanced Search  Database - CINAHL Plus | 27,333 |
| S78 | TI (“chat room*” or chatroom* or webchat* or “web chat*” or “social network*” or “social forum*”) OR AB (“chat room*” or chatroom* or webchat* or “web chat*” or “social network*” or “social forum*”) | Expanders - Apply equivalent subjects  Search modes - Boolean/Phrase | Interface - EBSCOhost Research Databases  Search Screen - Advanced Search  Database - CINAHL Plus | 13,034 |
| S77 | TI (internet or online or virtual or remote or telephon*) OR AB (internet or online or virtual or remote or telephon*) | Expanders - Apply equivalent subjects  Search modes - Boolean/Phrase | Interface - EBSCOhost Research Databases  Search Screen - Advanced Search  Database - CINAHL Plus | 179,987 |
| S76 | TI (ehealth or e-health or mhealth or m-health or mHealth or mCare or mcare) OR AB (ehealth or e-health or mhealth or m-health or mHealth or mCare or mcare) | Expanders - Apply equivalent subjects  Search modes - Boolean/Phrase | Interface - EBSCOhost Research Databases  Search Screen - Advanced Search  Database - CINAHL Plus | 6,176 |
| S75 | TI (email* or e-mail* or e-mailing or “electronic mail*”) OR AB (email* or e-mail* or e-mailing or “electronic mail*”) | Expanders - Apply equivalent subjects  Search modes - Boolean/Phrase | Interface - EBSCOhost Research Databases  Search Screen - Advanced Search  Database - CINAHL Plus | 12,252 |
| S74 | TI (“text messag*” or texting or text* or “short message service” or SMS) OR AB (“text messag*” or texting or text* or “short message service” or SMS) | Expanders - Apply equivalent subjects  Search modes - Boolean/Phrase | Interface - EBSCOhost Research Databases  Search Screen - Advanced Search  Database - CINAHL Plus | 48,630 |
| S73 | TI (“mobile app*” or “moblie application*” or software or “portable software app*” or “portable mobile application*” or pda or pdas or “mobile game*” or “personal digital assistan*”) OR AB (“mobile app*” or “moblie application*” or software or “portable software app*” or “portable mobile application*” or pda or pdas or “mobile game*” or “personal digital assistan*”) | Expanders - Apply equivalent subjects  Search modes - Boolean/Phrase | Interface - EBSCOhost Research Databases  Search Screen - Advanced Search  Database - CINAHL Plus | 58,932 |
| S72 | TI (“wireless communication*” or “mobile communication*” or “wireless technolog*” or “mobile techonolog*”) OR AB (“wireless communication*” or “mobile communication*” or “wireless technolog*” or “mobile techonolog*”) | Expanders - Apply equivalent subjects  Search modes - Boolean/Phrase | Interface - EBSCOhost Research Databases  Search Screen - Advanced Search  Database - CINAHL Plus | 702 |
| S71 | TI (microcomputer* or "micro computer*" or micro-computer* or tablet* or ipad) OR AB (microcomputer* or "micro computer*" or micro-computer* or tablet* or ipad) | Expanders - Apply equivalent subjects  Search modes - Boolean/Phrase | Interface - EBSCOhost Research Databases  Search Screen - Advanced Search  Database - CINAHL Plus | 13,196 |
| S70 | TI ("handheld computer*" or "hand held computer*" or smartphone* or "smart phone*") OR AB ("handheld computer*" or "hand held computer*" or smartphone* or "smart phone*") | Expanders - Apply equivalent subjects  Search modes - Boolean/Phrase | Interface - EBSCOhost Research Databases  Search Screen - Advanced Search  Database - CINAHL Plus | 8,286 |
| S69 | TI ("cell phone*" or cellphone* or telephone* or "cellular phone*" or "cellular telephone*" or "mobile telephone*" or "mobile phone*") OR AB ("cell phone*" or cellphone* or telephone* or "cellular phone*" or "cellular telephone*" or "mobile telephone*" or "mobile phone*") | Expanders - Apply equivalent subjects  Search modes - Boolean/Phrase | Interface - EBSCOhost Research Databases  Search Screen - Advanced Search  Database - CINAHL Plus | 38,134 |
| S68 | (MH "Blogs") | Expanders - Apply equivalent subjects  Search modes - Boolean/Phrase | Interface - EBSCOhost Research Databases  Search Screen - Advanced Search  Database - CINAHL Plus | 3,699 |
| S67 | (MH "Social Media+") | Expanders - Apply equivalent subjects  Search modes - Boolean/Phrase | Interface - EBSCOhost Research Databases  Search Screen - Advanced Search  Database - CINAHL Plus | 18,535 |
| S66 | (MH "Internet+") | Expanders - Apply equivalent subjects  Search modes - Boolean/Phrase | Interface - EBSCOhost Research Databases  Search Screen - Advanced Search  Database - CINAHL Plus | 160,039 |
| S65 | (MH "Reminder Systems") | Expanders - Apply equivalent subjects  Search modes - Boolean/Phrase | Interface - EBSCOhost Research Databases  Search Screen - Advanced Search  Database - CINAHL Plus | 3,037 |
| S64 | (MH "Text Messaging+") OR (MH "Instant Messaging") | Expanders - Apply equivalent subjects  Search modes - Boolean/Phrase | Interface - EBSCOhost Research Databases  Search Screen - Advanced Search  Database - CINAHL Plus | 3,932 |
| S63 | (MH "Wireless Communications") | Expanders - Apply equivalent subjects  Search modes - Boolean/Phrase | Interface - EBSCOhost Research Databases  Search Screen - Advanced Search  Database - CINAHL Plus | 12,130 |
| S62 | (MH "Telephone+") OR (MH "Telephone Information Services") OR (MH "Cellular Phone+") | Expanders - Apply equivalent subjects  Search modes - Boolean/Phrase | Interface - EBSCOhost Research Databases  Search Screen - Advanced Search  Database - CINAHL Plus | 28,988 |
| S61 | (MH "Exercise+") | Expanders - Apply equivalent subjects  Search modes - Boolean/Phrase | Interface - EBSCOhost Research Databases  Search Screen - Advanced Search  Database - CINAHL Plus | 123,528 |
| S60 | (MH "Diet+") | Expanders - Apply equivalent subjects  Search modes - Boolean/Phrase | Interface - EBSCOhost Research Databases  Search Screen - Advanced Search  Database - CINAHL Plus | 131,443 |
| S59 | (MH "Decision Making+") | Expanders - Apply equivalent subjects  Search modes - Boolean/Phrase | Interface - EBSCOhost Research Databases  Search Screen - Advanced Search  Database - CINAHL Plus | 137,229 |
| S58 | TI (decision* N2 (shared or support* or aid or adis or making)) OR AB (decision* N2 (shared or support* or aid or adis or making)) | Expanders - Apply equivalent subjects  Search modes - Boolean/Phrase | Interface - EBSCOhost Research Databases  Search Screen - Advanced Search  Database - CINAHL Plus | 83,017 |
| S57 | TI (involv* or participat* or collaborat* or empower* or enabl* or engag* or partner*) OR AB (involv* or participat* or collaborat* or empower* or enabl* or engag* or partner*) | Expanders - Apply equivalent subjects  Search modes - Boolean/Phrase | Interface - EBSCOhost Research Databases  Search Screen - Advanced Search  Database - CINAHL Plus | 816,364 |
| S56 | TI "patient participation" OR AB "patient participation" | Expanders - Apply equivalent subjects  Search modes - Boolean/Phrase | Interface - EBSCOhost Research Databases  Search Screen - Advanced Search  Database - CINAHL Plus | 1,592 |
| S55 | (MH "Consumer Participation") | Expanders - Apply equivalent subjects  Search modes - Boolean/Phrase | Interface - EBSCOhost Research Databases  Search Screen - Advanced Search  Database - CINAHL Plus | 22,001 |
| S54 | TI (educational or instructional or teaching) N1 (material* or leaflet* or booklet* or toolkit* or resource*)) OR AB (educational or instructional or teaching) N1 (material* or booklet* or toolkit* or resource*)) | Expanders - Apply equivalent subjects  Search modes - Boolean/Phrase | Interface - EBSCOhost Research Databases  Search Screen - Advanced Search  Database - CINAHL Plus | 5,907 |
| S53 | (MH "Bibliotherapy") | Expanders - Apply equivalent subjects  Search modes - Boolean/Phrase | Interface - EBSCOhost Research Databases  Search Screen - Advanced Search  Database - CINAHL Plus | 421 |
| S52 | (MH "Pamphlets") | Expanders - Apply equivalent subjects  Search modes - Boolean/Phrase | Interface - EBSCOhost Research Databases  Search Screen - Advanced Search  Database - CINAHL Plus | 3,570 |
| S51 | (MH "Teaching Materials+") | Expanders - Apply equivalent subjects  Search modes - Boolean/Phrase | Interface - EBSCOhost Research Databases  Search Screen - Advanced Search  Database - CINAHL Plus | 128,380 |
| S50 | TI nurse N2 educator* OR AB nurse N2 educator* | Expanders - Apply equivalent subjects  Search modes - Boolean/Phrase | Interface - EBSCOhost Research Databases  Search Screen - Advanced Search  Database - CINAHL Plus | 6,522 |
| S49 | TI (patient N2 (educat* or advice or advis* or instruct* or train* or coach*)) OR AB (patient N2 (educat* or advice or advis* or instruct* or train* or coach*)) | Expanders - Apply equivalent subjects  Search modes - Boolean/Phrase | Interface - EBSCOhost Research Databases  Search Screen - Advanced Search  Database - CINAHL Plus | 39,007 |
| S48 | TI ((patient or client or "consumer health") W1 (education or information)) OR AB ((patient or client or "consumer health") W1 (education or information)) | Expanders - Apply equivalent subjects  Search modes - Boolean/Phrase | Interface - EBSCOhost Research Databases  Search Screen - Advanced Search  Database - CINAHL Plus | 22,331 |
| S47 | (MH "Patient Education+") | Expanders - Apply equivalent subjects  Search modes - Boolean/Phrase | Interface - EBSCOhost Research Databases  Search Screen - Advanced Search  Database - CINAHL Plus | 82,704 |
| S46 | (MH "Recovery") | Expanders - Apply equivalent subjects  Search modes - Boolean/Phrase | Interface - EBSCOhost Research Databases  Search Screen - Advanced Search  Database - CINAHL Plus | 35,761 |
| S45 | TI "interprersonal communication" OR AB "interpersonal communication" | Expanders - Apply equivalent subjects  Search modes - Boolean/Phrase | Interface - EBSCOhost Research Databases  Search Screen - Advanced Search  Database - CINAHL Plus | 907 |
| S44 | (MH "Communication+") | Expanders - Apply equivalent subjects  Search modes - Boolean/Phrase | Interface - EBSCOhost Research Databases  Search Screen - Advanced Search  Database - CINAHL Plus | 302,598 |
| S43 | (MH "Problem Solving+") | Expanders - Apply equivalent subjects  Search modes - Boolean/Phrase | Interface - EBSCOhost Research Databases  Search Screen - Advanced Search  Database - CINAHL Plus | 12,746 |
| S42 | (MH "Meditation") | Expanders - Apply equivalent subjects  Search modes - Boolean/Phrase | Interface - EBSCOhost Research Databases  Search Screen - Advanced Search  Database - CINAHL Plus | 5,665 |
| S41 | (MH "Mindfulness") | Expanders - Apply equivalent subjects  Search modes - Boolean/Phrase | Interface - EBSCOhost Research Databases  Search Screen - Advanced Search  Database - CINAHL Plus | 6,316 |
| S40 | (MH "Coping+") | Expanders - Apply equivalent subjects  Search modes - Boolean/Phrase | Interface - EBSCOhost Research Databases  Search Screen - Advanced Search  Database - CINAHL Plus | 39,189 |
| S39 | (MH "Adaptation, Psychological+") | Expanders - Apply equivalent subjects  Search modes - Boolean/Phrase | Interface - EBSCOhost Research Databases  Search Screen - Advanced Search  Database - CINAHL Plus | 37,945 |
| S38 | TI "psychoeducat*" OR AB "psychoeducat*" | Expanders - Apply equivalent subjects  Search modes - Boolean/Phrase | Interface - EBSCOhost Research Databases  Search Screen - Advanced Search  Database - CINAHL Plus | 3,314 |
| S37 | (MH "Psychoeducation") | Expanders - Apply equivalent subjects  Search modes - Boolean/Phrase | Interface - EBSCOhost Research Databases  Search Screen - Advanced Search  Database - CINAHL Plus | 3,513 |
| S36 | (MH "Behavior Therapy+") | Expanders - Apply equivalent subjects  Search modes - Boolean/Phrase | Interface - EBSCOhost Research Databases  Search Screen - Advanced Search  Database - CINAHL Plus | 37,960 |
| S35 | (MH "Behavior Modification+") | Expanders - Apply equivalent subjects  Search modes - Boolean/Phrase | Interface - EBSCOhost Research Databases  Search Screen - Advanced Search  Database - CINAHL Plus | 56,415 |
| S34 | TI (CBT or "cognitive therap*" or "cognitive behav*") OR AB (CBT or "cognitive therap*" or "cognitive behav*") | Expanders - Apply equivalent subjects  Search modes - Boolean/Phrase | Interface - EBSCOhost Research Databases  Search Screen - Advanced Search  Database - CINAHL Plus | 18,892 |
| S33 | TI ((behaviour* or behavior*) W1 (manag* or modif* or therap*)) OR AB ((behaviour* or behavior*) W1 (manag* or modif* or therap*)) | Expanders - Apply equivalent subjects  Search modes - Boolean/Phrase | Interface - EBSCOhost Research Databases  Search Screen - Advanced Search  Database - CINAHL Plus | 17,256 |
| S32 | TI ("skill training" or "coping skill*" or empower*) OR AB ("skill training" or "coping skill*" or empower*) | Expanders - Apply equivalent subjects  Search modes - Boolean/Phrase | Interface - EBSCOhost Research Databases  Search Screen - Advanced Search  Database - CINAHL Plus | 28,257 |
| S31 | TI ("psychological adaptation" or "adaptive behav*") OR AB ("psychological adaptation" or "adaptive behav*") | Expanders - Apply equivalent subjects  Search modes - Boolean/Phrase | Interface - EBSCOhost Research Databases  Search Screen - Advanced Search  Database - CINAHL Plus | 1,765 |
| S30 | TI ("emotional adjustment" or "psychological adjustment" or "social adjustment") OR AB ("emotional adjustment" or "psychological adjustment" or "social adjustment") | Expanders - Apply equivalent subjects  Search modes - Boolean/Phrase | Interface - EBSCOhost Research Databases  Search Screen - Advanced Search  Database - CINAHL Plus | 2,853 |
| S29 | TI "motivational therapy" OR AB "motivational therapy" | Expanders - Apply equivalent subjects  Search modes - Boolean/Phrase | Interface - EBSCOhost Research Databases  Search Screen - Advanced Search  Database - CINAHL Plus | 15 |
| S28 | (MH "Psychotherapy+") OR (MH "Psychotherapy, Brief+") OR (MH "Psychotherapy, Group+") | Expanders - Apply equivalent subjects  Search modes - Boolean/Phrase | Interface - EBSCOhost Research Databases  Search Screen - Advanced Search  Database - CINAHL Plus | 212,314 |
| S27 | (MH "Cognitive Therapy+") | Expanders - Apply equivalent subjects  Search modes - Boolean/Phrase | Interface - EBSCOhost Research Databases  Search Screen - Advanced Search  Database - CINAHL Plus | 26,215 |
| S26 | (MH "Motivational Interviewing") | Expanders - Apply equivalent subjects  Search modes - Boolean/Phrase | Interface - EBSCOhost Research Databases  Search Screen - Advanced Search  Database - CINAHL Plus | 3,774 |
| S25 | TI "health trainer* OR AB "health trainer*" | Expanders - Apply equivalent subjects  Search modes - SmartText Searching | Interface - EBSCOhost Research Databases  Search Screen - Advanced Search  Database - CINAHL Plus | 1,289 |
| S24 | TI (befriend* or coach* or mentor* or buddy or buddies) OR AB (befriend* or coach* or mentor* or buddy or buddies) | Expanders - Apply equivalent subjects  Search modes - Boolean/Phrase | Interface - EBSCOhost Research Databases  Search Screen - Advanced Search  Database - CINAHL Plus | 29,089 |
| S23 | TI "social support*" OR AB "social support*" | Expanders - Apply equivalent subjects  Search modes - Boolean/Phrase | Interface - EBSCOhost Research Databases  Search Screen - Advanced Search  Database - CINAHL Plus | 30,009 |
| S22 | TI ("expert patient*" or "virtual communit*" or "online communit*") OR AB ("expert patient*" or "virtual communit*" or "online communit*") | Expanders - Apply equivalent subjects  Search modes - Boolean/Phrase | Interface - EBSCOhost Research Databases  Search Screen - Advanced Search  Database - CINAHL Plus | 1,452 |
| S21 | TI ((mutual or telephone or online or virtual) W1 support) OR AB ((mutual or telephone or online or virtual) W1 support) | Expanders - Apply equivalent subjects  Search modes - Boolean/Phrase | Interface - EBSCOhost Research Databases  Search Screen - Advanced Search  Database - CINAHL Plus | 2,357 |
| S20 | TI (peer W1 (support or advice or advis* or monitor* or intervention* or train* or instruct* or consult* or assist* or educat* or information)) OR AB (peer W1 (support or advice or advis* or monitor* or intervention* or train* or instruct* or consult* or assist* or educat* or information)) | Expanders - Apply equivalent subjects  Search modes - Boolean/Phrase | Interface - EBSCOhost Research Databases  Search Screen - Advanced Search  Database - CINAHL Plus | 7,512 |
| S19 | TI (group W1 (support or advice or advis* or monitor* or intervention* or train* or instruct* or consult* or assist* or educat* or information)) OR AB (group W1 (support or advice or advis* or monitor* or intervention* or train* or instruct* or consult* or assist* or educat* or information)) | Expanders - Apply equivalent subjects  Search modes - Boolean/Phrase | Interface - EBSCOhost Research Databases  Search Screen - Advanced Search  Database - CINAHL Plus | 16,939 |
| S18 | (MH "Support Groups+") | Expanders - Apply equivalent subjects  Search modes - Boolean/Phrase | Interface - EBSCOhost Research Databases  Search Screen - Advanced Search  Database - CINAHL Plus | 11,846 |
| S17 | (MH "Support, Psychosocial+") | Expanders - Apply equivalent subjects  Search modes - Boolean/Phrase | Interface - EBSCOhost Research Databases  Search Screen - Advanced Search  Database - CINAHL Plus | 93,154 |
| S16 | S1 OR S2 OR S3 OR S4 OR S5 OR S6 OR S7 OR S8 OR S9 OR S10 OR S11 OR S12 OR S13 OR S14 OR S15 | Expanders - Apply equivalent subjects  Search modes - Boolean/Phrase | Interface - EBSCOhost Research Databases  Search Screen - Advanced Search  Database - CINAHL Plus | 210,942 |
| S15 | (MH "Self Medication") | Expanders - Apply equivalent subjects  Search modes - Boolean/Phrase | Interface - EBSCOhost Research Databases  Search Screen - Advanced Search  Database - CINAHL Plus | 2,080 |
| S14 | TI (self initiat* or self-initiat* or selfregulat* or self regulat* or self-regulat*) OR AB (self initiat* or self-initiat* or selfregulat* or self regulat* or self-regulat*) | Expanders - Apply equivalent subjects  Search modes - Boolean/Phrase | Interface - EBSCOhost Research Databases  Search Screen - Advanced Search  Database - CINAHL Plus | 8,861 |
| S13 | TI (selfrecover* or self recover* or self-recover*) OR AB (selfrecover* or self recover* or self-recover*) | Expanders - Apply equivalent subjects  Search modes - Boolean/Phrase | Interface - EBSCOhost Research Databases  Search Screen - Advanced Search  Database - CINAHL Plus | 1,525 |
| S12 | TI (selftreat* or self treat* or self-treat* or selfmedicat* or self medicat* or self-medicat* or selfcure* or self cure* or self-cure* or selfcuring or self curing or self-curing) OR AB (selftreat* or self treat* or self-treat* or selfmedicat* or self medicat* or self-medicat* or selfcure* or self cure* or self-cure* or selfcuring or self curing or self-curing) | Expanders - Apply equivalent subjects  Search modes - Boolean/Phrase | Interface - EBSCOhost Research Databases  Search Screen - Advanced Search  Database - CINAHL Plus | 13,436 |
| S11 | TI (selfhelp* or self help* or self-help*) OR AB (selfhelp* or self help* or self-help*) | Expanders - Apply equivalent subjects  Search modes - Boolean/Phrase | Interface - EBSCOhost Research Databases  Search Screen - Advanced Search  Database - CINAHL Plus | 7,997 |
| S10 | TI (selfmonitor* or self monitor* or self monitor* or self policing or selfpolicing or self-policing or selfsupervis* or self supervis* or self-supervis*) OR AB (selfmonitor* or self monitor* or self monitor* or self policing or selfpolicing or self-policing or selfsupervis* or self supervis* or self-supervis*) | Expanders - Apply equivalent subjects  Search modes - Boolean/Phrase | Interface - EBSCOhost Research Databases  Search Screen - Advanced Search  Database - CINAHL Plus | 6,354 |
| S9 | TI (selfreport* or self report* or self-report*) OR AB (selfreport* or self report* or self-report*) | Expanders - Apply equivalent subjects  Search modes - Boolean/Phrase | Interface - EBSCOhost Research Databases  Search Screen - Advanced Search  Database - CINAHL Plus | 95,916 |
| S8 | TI (selfassess* or self assess* or self-assess* or selfdiagnos* or self diagnos* or self-diagnos*) OR AB (selfassess* or self assess* or self-assess* or selfdiagnos* or self diagnos* or self-diagnos*) | Expanders - Apply equivalent subjects  Search modes - Boolean/Phrase | Interface - EBSCOhost Research Databases  Search Screen - Advanced Search  Database - CINAHL Plus | 30,916 |
| S7 | TI (self manag* or selfmanag* or self-manag*) OR AB (self manag* or selfmanag* or self-manag*) | Expanders - Apply equivalent subjects  Search modes - Boolean/Phrase | Interface - EBSCOhost Research Databases  Search Screen - Advanced Search  Database - CINAHL Plus | 21,804 |
| S6 | TI (self-care* or selfcaring or selfcare* or self caring) OR AB (self-care* or self caring or selfcare* or selfcaring) | Expanders - Apply equivalent subjects  Search modes - Boolean/Phrase | Interface - EBSCOhost Research Databases  Search Screen - Advanced Search  Database - CINAHL Plus | 18,639 |
| S5 | (MH "Self Care+") | Expanders - Apply equivalent subjects  Search modes - Boolean/Phrase | Interface - EBSCOhost Research Databases  Search Screen - Advanced Search  Database - CINAHL Plus | 55,961 |
| S4 | S2 NOT S3 | Expanders - Apply equivalent subjects  Search modes - Boolean/Phrase | Interface - EBSCOhost Research Databases  Search Screen - Advanced Search  Database - CINAHL Plus | 3,819 |
| S3 | TI (self-administer* N2 (questionnaire* or survey* or interview*) OR AB (self-administer* N2 (questionnaire* or survey* or interview*)) | Expanders - Apply equivalent subjects  Search modes - Boolean/Phrase | Interface - EBSCOhost Research Databases  Search Screen - Advanced Search  Database - CINAHL Plus | 12,063 |
| S2 | TI self-adminsiter* OR AB self-administer* | Expanders - Apply equivalent subjects  Search modes - Boolean/Phrase | Interface - EBSCOhost Research Databases  Search Screen - Advanced Search  Database - CINAHL Plus | 15,806 |
| S1 | (MH "Self Administration+") | Expanders - Apply equivalent subjects  Search modes - Boolean/Phrase | Interface - EBSCOhost Research Databases  Search Screen - Advanced Search  Database - CINAHL Plus | 6,155 |

**Database(s): Ovid EBM Reviews** - Cochrane Database of Systematic Reviews 2005 to November 18, 2021, EBM Reviews - ACP Journal Club 1991 to October 2021, EBM Reviews - Database of Abstracts of Reviews of Effects 1st Quarter 2016, EBM Reviews - Cochrane Clinical Answers October 2021, EBM Reviews - Cochrane Central Register of Controlled Trials October 2021, EBM Reviews - Cochrane Methodology Register 3rd Quarter 2012, EBM Reviews - Health Technology Assessment 4th Quarter 2016, EBM Reviews - NHS Economic Evaluation Database 1st Quarter 2016

**Date searched: 22 November 2021**

**Records retrieved: 30**

**Search Strategy:**

| **#** | **Searches** | **Results** |
| --- | --- | --- |
| 1 | self administer*.ti,ab. | 6277 |
| 2 | (self administer* adj2 (questionnaire* or survey* or interview*)).ti,ab. | 2174 |
| 3 | 1 not 2 | 4103 |
| 4 | (self care* or selfcaring or selfcare* or self-care* or self caring or self-caring).ti,ab. | 5706 |
| 5 | (self manag* or selfmanag* or self-manag*).ti,ab. | 9543 |
| 6 | (selfassess* or self-assess* or self assess* or selfdiagnos* or self-diagnos* or self diagnos*).ti,ab. | 4182 |
| 7 | (selfmonitor* or self monitor* or self monitor* or self policing or selfpolicing or self-policing or selfsupervis* or self supervis* or self-supervis*).ti,ab. | 4495 |
| 8 | (selfhelp* or self help* or self-help*).ti,ab. | 3430 |
| 9 | (selftreat* or self treat* or self-treat* or selfmedicat* or self medicat* or self-medicat* or selfcure* or self cure* or self-cure* or selfcuring or self curing or self-curing).ti,ab. | 759 |
| 10 | (selfrecover* or self recover* or self-recover*).ti,ab. | 9 |
| 11 | (self initiat* or self-initiat* or selfregulat* or self regulat* or self-regulat*).ti,ab. | 2687 |
| 12 | self administration.ti,ab. | 1198 |
| 13 | 3 or 4 or 5 or 6 or 7 or 8 or 9 or 10 or 11 or 12 | 33129 |
| 14 | social support*.ti,ab. | 6113 |
| 15 | social support.ti,ab. | 6014 |
| 16 | (group adj (support or advice or advis* or monitor* or intervention* or train* or instruct* or consult* or assist* or educat* or information)).ti,ab. | 19643 |
| 17 | (peer adj (support or advice or advis* or monitor* or intervention* or train* or instruct* or consult* or assist* or educat* or information or mentor*)).ti,ab. | 2335 |
| 18 | ((mutual or telephone or online or virtual or digital) adj support).ti,ab. | 1109 |
| 19 | (expert patient* or virtual communit* or online communit*).ti,ab. | 237 |
| 20 | (befriend* or coach* or mentor* or buddy or buddies).ti,ab. | 7813 |
| 21 | health trainer*.ti,ab. | 28 |
| 22 | psychotherapy.ti,ab. | 7322 |
| 23 | group therapy.ti,ab. | 2512 |
| 24 | psychoeducat*.ti,ab. | 3834 |
| 25 | cognitive therapy.ti,ab. | 2272 |
| 26 | cognitive behavioural therapy.ti,ab. | 3065 |
| 27 | cognitive behavioral therapy.ti,ab. | 7462 |
| 28 | motivational therapy.ti,ab. | 30 |
| 29 | motivational interviewing.ti,ab. | 3801 |
| 30 | (skill training or coping skill* or empower*).ti,ab. | 6203 |
| 31 | ((psychological or emotional or psychosocial) adj2 (adjustment or adaptation)).ti,ab. | 887 |
| 32 | adaptive behav*.ti,ab. | 539 |
| 33 | ((behaviour* or behavior*) adj (manag* or therap* or modif*)).ti,ab. | 17449 |
| 34 | mindfulness.ti,ab. | 6297 |
| 35 | meditation.ti,ab. | 2957 |
| 36 | problem solving.ti,ab. | 4741 |
| 37 | communication.ti,ab. | 17667 |
| 38 | recovery.ti,ab. | 60540 |
| 39 | (CBT or cognitive therap* or cognitive behav*).ti,ab. | 23055 |
| 40 | patient education.ti,ab. | 3775 |
| 41 | ((patient or consumer health) adj (education or information)).ti,ab. | 5516 |
| 42 | (patient adj2 (educat* or advice or advis* or instruct* or train* or coach*)).ti,ab. | 7425 |
| 43 | (nurse adj educator*).ti,ab. | 180 |
| 44 | ((teaching or educational or informational) adj (material* or pamplet* or resource*)).ti,ab. | 2339 |
| 45 | bibliotherapy.ti,ab. | 284 |
| 46 | patient participation.ti,ab. | 601 |
| 47 | (involv* or participat* or collaborat* or empower* or enabl* or engag* or partner*).ti,ab. | 265152 |
| 48 | (decision* adj2 (shared or support* or aid or adis or making)).ti,ab. | 16255 |
| 49 | decision making.ti,ab. | 12325 |
| 50 | diet.ti,ab. | 47324 |
| 51 | exercise.ti,ab. | 91781 |
| 52 | telemedicine.ti,ab. | 1982 |
| 53 | (telemedicine or teleconsultation or telecare or telenursing or telemonitor* or telehealth or ehealth).ti,ab. | 5173 |
| 54 | ((telephone or remote or phone or mobile or digital or virtual or online) adj2 (follow* or support* or consult* or advice or advis* or intervention* or instruct* or assist* or educat* or information or monitor*)).ti,ab. | 18500 |
| 55 | mobile phone*.ti,ab. | 2948 |
| 56 | (cell phone* or cellphone* or telephone* or cellular phone* or cellular telephone* or mobile telephone* or mobile phone*).ti,ab. | 25302 |
| 57 | (handheld computer* or hand held computer* or smartphone* or smart phone*).ti,ab. | 5560 |
| 58 | telephone.ti,ab. | 21685 |
| 59 | (microcomputer* or micro computer* or micro-computer* or tablet* or ipad).ti,ab. | 48501 |
| 60 | (iphone or android* or blackberry).ti,ab. | 1099 |
| 61 | (wireless communication* or mobile communication* or wireless technolog* or mobile techonolog*).ti,ab. | 150 |
| 62 | (mobile app* or moblie application* or software or portable software app* or portable mobile application* or pda or pdas or mobile game* or personal digital assistan*).ti,ab. | 21535 |
| 63 | text messaging.ti,ab. | 1648 |
| 64 | reminder system*.ti,ab. | 352 |
| 65 | (text messag* or texting or text* or short message service or SMS).ti,ab. | 15928 |
| 66 | (text messag* or texting or text* or short message service* or SMS or e-mail* or email* or electronic mail*).ti,ab. | 20881 |
| 67 | (ehealth or e-health or mhealth or m-health or mHealth or m-Health or mCare or mcare or m-care or m-Care).ti,ab. | 2810 |
| 68 | (internet or online or virtual or remote or telephon* or website* or web site*).ti,ab. | 62483 |
| 69 | blog*.ti,ab. | 164 |
| 70 | social media.ti,ab. | 1600 |
| 71 | (chat room* or chatroom* or webchat* or web chat* or social network* or social forum*).ti,ab. | 1811 |
| 72 | or/14-71 | 564722 |
| 73 | (schoolchild* or school child* or teen* or teenager* or young person or young people or young adult* or emerging adult* or youth* or adolescen* child or children or student* or juvenile* or youngster* or kid or kids or pediatric or paediatric).ti,ab. | 180817 |
| 74 | sickle cell*.ti,ab. | 2300 |
| 75 | (sickle cell anaemia or sickle cell anemia).ti,ab. | 685 |
| 76 | (sickle cell disease* or sickle cell disorder* or sickle cell syndrome).ti,ab. | 1589 |
| 77 | (SCD or SCA or HbSS or HbSC).ti,ab. | 2024 |
| 78 | (haemoglobin S or hemoglobin S or haemoglobin SC or hemoglobin SC).ti,ab. | 80 |
| 79 | 74 or 75 or 76 or 77 or 78 | 3331 |
| 80 | 13 and 72 and 73 and 79 | 30 |
| 81 | remove duplicates from 80 | 30 |

**Database: Web of Science (All Databases)**

**Date searched: 12 November 2021**

**Date range searched: 1900 to present**

**Records retrieved: 157**

The same search strategy was used across **Applied Social Sciences Indexes and Abstracts (ProQuest)**

**Date searched: 12 November 2021**

**Date range searched: 1987 to present**

**Records retrieved: 156**

**Search Strategy:**

| # 28 | [**157**](https://apps.webofknowledge.com/summary.do?product=UA&doc=1&qid=136&SID=E1t3sem1IfBgUgnK7mF&search_mode=CombineSearches&update_back2search_link_param=yes) | #27 AND #26 AND #25 AND #5 |  |  |  |
| --- | --- | --- | --- | --- | --- |
|  | | | | |  |
| # 27 | [**85,927**](https://apps.webofknowledge.com/summary.do?product=UA&doc=1&qid=135&SID=E1t3sem1IfBgUgnK7mF&search_mode=AdvancedSearch&update_back2search_link_param=yes) | TI=("sickle cell" or "sickle cell disease*" or "sickle cell disorder*" or "sickle cell syndrome" or "sickle cell anaemia" or "sickle cell anemia" or SCD or SCA or HbSS or HbSC or HBSS or HBSC or "haemoglobin S" or "hemoglobin S" or "haemoglobin SC" or "hemoglobin SC") OR AB=("sickle cell" or "sickle cell disease*" or "sickle cell disorder*" or "sickle cell syndrome" or "sickle cell anaemia" or "sickle cell anemia" or SCD or SCA or HbSS or HbSC or HBSS or HBSC or "haemoglobin S" or "hemoglobin S" or "haemoglobin SC" or "hemoglobin SC") |  |  |  |
|  | | | | |  |
| # 26 | [**5,916,967**](https://apps.webofknowledge.com/summary.do?product=UA&doc=1&qid=134&SID=E1t3sem1IfBgUgnK7mF&search_mode=AdvancedSearch&update_back2search_link_param=yes) | TI=(child* or schoolchild* or "school child*" or teen* or teenager* or "young person" or "young people" or "young adult" or "emerging adult" or youth* or adolescen* or student* or juvenile* or youngster* or kid* or pediatric or paediatric) OR AB=(child* or schoolchild* or "school child*" or teen* or teenager* or "young person" or "young people" or "young adult" or "emerging adult" or youth* or adolescen* or student* or juvenile* or youngster* or kid* or pediatric or paediatric) |  |  |  |
|  | | | | |  |
| # 25 | [**18,414,283**](https://apps.webofknowledge.com/summary.do?product=UA&doc=1&qid=133&SID=E1t3sem1IfBgUgnK7mF&search_mode=CombineSearches&update_back2search_link_param=yes) | #24 OR #23 OR #22 OR #21 OR #20 OR #19 OR #18 OR #17 OR #16 OR #15 OR #14 OR #13 OR #12 OR #11 OR #10 OR #9 OR #8 OR #7 OR #6 |  |  |  |
|  | | | | |  |
| # 24 | [**26,579**](https://apps.webofknowledge.com/summary.do?product=UA&doc=1&qid=132&SID=E1t3sem1IfBgUgnK7mF&search_mode=AdvancedSearch&update_back2search_link_param=yes) | TI=(blog*) OR AB=(blog*) |  |  |  |
|  | | | | |  |
| # 23 | [**18,689**](https://apps.webofknowledge.com/summary.do?product=UA&doc=1&qid=129&SID=E1t3sem1IfBgUgnK7mF&search_mode=AdvancedSearch&update_back2search_link_param=yes) | TI=(ehealth or e-health or eHealth or e-Health or mhealth or m-health or mHealth or m-Health or mcare of m-care of mCare or m-Care) OR AB=(ehealth or e-health or eHealth or e-Health or mhealth or m-health or mHealth or m-Health or mcare of m-care of mCare or m-Care) |  |  |  |
|  | | | | |  |
| # 22 | [**3,959,427**](https://apps.webofknowledge.com/summary.do?product=UA&doc=1&qid=128&SID=E1t3sem1IfBgUgnK7mF&search_mode=AdvancedSearch&update_back2search_link_param=yes) | TI=("social media" or chatroom* or "chat room*" or webchat* or "web chat*" or "social network*" or "social forum" or internet or online or virtual or remote or telephon*) OR AB=("social media" or chatroom* or "chat room*" or webchat* or "web chat*" or "social network*" or "social forum" or internet or online or virtual or remote or telephon*) |  |  |  |
|  | | | | |  |
| # 21 | [**7,334,666**](https://apps.webofknowledge.com/summary.do?product=UA&doc=1&qid=127&SID=E1t3sem1IfBgUgnK7mF&search_mode=AdvancedSearch&update_back2search_link_param=yes) | TI=("text messag*" or "reminder system*" or texting or text* or "short message service" or SMS or email* or e-mail* or e-mailing or "e mailing" or "electronic mail*" or website* or "web site*") OR AB=("text messag*" or "reminder system*" or texting or text* or "short message service" or SMS or email* or e-mail* or e-mailing or "e mailing" or "electronic mail*" or website* or "web site*") |  |  |  |
|  | | | | |  |
| # 20 | [**2,447,458**](https://apps.webofknowledge.com/summary.do?product=UA&doc=1&qid=126&SID=E1t3sem1IfBgUgnK7mF&search_mode=AdvancedSearch&update_back2search_link_param=yes) | TI=("wireless communication*" or "mobile communication*" or "wireless technolog*" or "mobile technolog*" or "computer mediated communication" or "mobile app*" or "mobile application*" or software or "portable software app*" or "portable mobile application*" or pda or pdas or "mobile game*" or "personal digital assistan*") OR AB=("wireless communication*" or "mobile communication*" or "wireless technolog*" or "mobile technolog*" or "computer mediated communication" or "mobile app*" or "mobile application*" or software or "portable software app*" or "portable mobile application*" or pda or pdas or "mobile game*" or "personal digital assistan*") |  |  |  |
|  | | | | |  |
| # 19 | [**1,861,013**](https://apps.webofknowledge.com/summary.do?product=UA&doc=1&qid=125&SID=E1t3sem1IfBgUgnK7mF&search_mode=AdvancedSearch&update_back2search_link_param=yes) | TI=("mobile phone*" or telephone or "cell phone*" or "cellular phone*" or "cellular telephone*" or "mobile telephone*" or "handheld computer*" or "hand held computer*" or smartphone* or "smart phone*" or microcomputer* or "micro computer*" or micro-computer* or tablet* or iPad* or ipad* or iPhone or iphone or android* or blackberry) OR AB=("mobile phone*" or telephone or "cell phone*" or "cellular phone*" or "cellular telephone*" or "mobile telephone*" or "handheld computer*" or "hand held computer*" or smartphone* or "smart phone*" or microcomputer* or "micro computer*" or micro-computer* or tablet* or iPad* or ipad* or iPhone or iphone or android* or blackberry) |  |  |  |
|  | | | | |  |
| # 18 | [**68,591**](https://apps.webofknowledge.com/summary.do?product=UA&doc=1&qid=124&SID=E1t3sem1IfBgUgnK7mF&search_mode=AdvancedSearch&update_back2search_link_param=yes) | TI=("telephone follow*" or "telephone support*" or "telephone consult*" or "telephone advice" or "telephone advis*" or "telephone intervention*" or "telephone instruct*" or "telephone assist*" or "telephone educat*" or "telephone information" or "telephone monitor*" or "remote follow*" or "remote support*" or "remote advice" or "remote advis*" or "remote intervention*" or "remote instruct" or "remote assist*" or "remote educat*" or "remote information" or "remote monitor*" or "phone follow*" or "phone support*" or "phone advice" or "phone advis*" or "phone consult*" or "phone intervention*" or "phone instruct*" or "phone assist*" or "phone educat*" or "phone information" or "phone monitor*") OR AB=("telephone follow*" or "telephone support*" or "telephone consult*" or "telephone advice" or "telephone advis*" or "telephone intervention*" or "telephone instruct*" or "telephone assist*" or "telephone educat*" or "telephone information" or "telephone monitor*" or "remote follow*" or "remote support*" or "remote advice" or "remote advis*" or "remote intervention*" or "remote instruct" or "remote assist*" or "remote educat*" or "remote information" or "remote monitor*" or "phone follow*" or "phone support*" or "phone advice" or "phone advis*" or "phone consult*" or "phone intervention*" or "phone instruct*" or "phone assist*" or "phone educat*" or "phone information" or "phone monitor*") |  |  |  |
|  | | | | |  |
| # 17 | [**43,148**](https://apps.webofknowledge.com/summary.do?product=UA&doc=1&qid=122&SID=E1t3sem1IfBgUgnK7mF&search_mode=AdvancedSearch&update_back2search_link_param=yes) | TI=(telemedicine or telecare or telenursing or teleconsultation or telemonitor* or telehealth or ehealth or "remote consult*") OR AB=(telemedicine or telecare or telenursing or teleconsultation or telemonitor* or telehealth or ehealth or "remote consult*") |  |  |  |
|  | | | | |  |
| # 16 | [**1,529,699**](https://apps.webofknowledge.com/summary.do?product=UA&doc=1&qid=121&SID=E1t3sem1IfBgUgnK7mF&search_mode=AdvancedSearch&update_back2search_link_param=yes) | TI=(diet or exercise) OR AB=(diet or exercise) |  |  |  |
|  | | | | |  |
| # 15 | [**676,250**](https://apps.webofknowledge.com/summary.do?product=UA&doc=1&qid=120&SID=E1t3sem1IfBgUgnK7mF&search_mode=AdvancedSearch&update_back2search_link_param=yes) | TI=(decision near/2 (shared or support* or aid or aids or making) ) OR AB=(decision near/2 (shared or support* or aid or aids or making) ) |  |  |  |
|  | | | | |  |
| # 14 | [**205,514**](https://apps.webofknowledge.com/summary.do?product=UA&doc=1&qid=119&SID=E1t3sem1IfBgUgnK7mF&search_mode=AdvancedSearch&update_back2search_link_param=yes) | TI=(patient near/2 ( involv* or participat* or collaborat* or empower* or enabl* or engag* or partner*) ) OR AB=(patient near/2 ( involv* or participat* or collaborat* or empower* or enabl* or engag* or partner*) ) |  |  |  |
|  | | | | |  |
| # 13 | [**791**](https://apps.webofknowledge.com/summary.do?product=UA&doc=1&qid=118&SID=E1t3sem1IfBgUgnK7mF&search_mode=AdvancedSearch&update_back2search_link_param=yes) | TI=(informational near/2 (material* or leaflet* or booklet* or toolkit*) ) OR AB=(informational near/2 (material* or leaflet* or booklet* or toolkit*) ) |  |  |  |
|  | | | | |  |
| # 12 | [**12,666**](https://apps.webofknowledge.com/summary.do?product=UA&doc=1&qid=117&SID=E1t3sem1IfBgUgnK7mF&search_mode=AdvancedSearch&update_back2search_link_param=yes) | TI=(educational near/2 (material* or leaflet* or booklet* or toolkit*) ) OR AB=(educational near/2 (material* or leaflet* or booklet* or toolkit*) ) |  |  |  |
|  | | | | |  |
| # 11 | [**18,289**](https://apps.webofknowledge.com/summary.do?product=UA&doc=1&qid=116&SID=E1t3sem1IfBgUgnK7mF&search_mode=AdvancedSearch&update_back2search_link_param=yes) | TI=(teaching near/2 (material* or leaflet* or booklet* or toolkit*) ) OR AB=(teaching near/2 (material* or leaflet* or booklet* or toolkit*) ) |  |  |  |
|  | | | | |  |
| # 10 | [**40,808**](https://apps.webofknowledge.com/summary.do?product=UA&doc=1&qid=115&SID=E1t3sem1IfBgUgnK7mF&search_mode=AdvancedSearch&update_back2search_link_param=yes) | TI=(nurse near/2 educat*) OR AB=(nurse near/2 educat*) |  |  |  |
|  | | | | |  |
| # 9 | [**26,557**](https://apps.webofknowledge.com/summary.do?product=UA&doc=1&qid=114&SID=E1t3sem1IfBgUgnK7mF&search_mode=AdvancedSearch&update_back2search_link_param=yes) | TI=("patient educat*" or "patient advice" or "patient instruct*" or "patient advis*" or "patient train*" or "patient coach*") OR AB=("patient educat*" or "patient advice" or "patient instruct*" or "patient advis*" or "patient train*" or "patient coach*") |  |  |  |
|  | | | | |  |
| # 8 | [**5,111,446**](https://apps.webofknowledge.com/summary.do?product=UA&doc=1&qid=113&SID=E1t3sem1IfBgUgnK7mF&search_mode=AdvancedSearch&update_back2search_link_param=yes) | TI=("motivational interviewing" or "skill training" or "coping skill*" or empower* or mindfulness or meditation or "problem solving" or "interpersonal communication" or communication or recovery or "behaviour manag*" or "behaviour modifi*" or "behaviour therap*" or "behavior manag*" or "behavior modif*" or "behavior therap*") OR AB=("motivational interviewing" or "skill training" or "coping skill*" or empower* or mindfulness or meditation or "problem solving" or "interpersonal communication" or communication or recovery or "behaviour manag*" or "behaviour modifi*" or "behaviour therap*" or "behavior manag*" or "behavior modif*" or "behavior therap*") |  |  |  |
|  | | | | |  |
| # 7 | [**94,393**](https://apps.webofknowledge.com/summary.do?product=UA&doc=1&qid=112&SID=E1t3sem1IfBgUgnK7mF&search_mode=AdvancedSearch&update_back2search_link_param=yes) | TI=("cognitive therap*" or "cognitive behavioural therap*" or "cognitive behavioral therap*" or psychoeducat* or "motivational therap*" or "psychological adjustment" or "emotional adjustment" or "psychosocial adjustment" or "psychological adaptation" or "psychosocial adaptation" or "adaptive behav*" or "behaviour therap*" or "behavior therap*" or CBT or "cognitive therap*" or "cognitive behav*" ) OR AB=("cognitive therap*" or "cognitive behavioural therap*" or "cognitive behavioral therap*" or psychoeducat* or "motivational therap*" or "psychological adjustment" or "emotional adjustment" or "psychosocial adjustment" or "psychological adaptation" or "psychosocial adaptation" or "adaptive behav*" or "behaviour therap*" or "behavior therap*" or CBT or "cognitive therap*" or "cognitive behav*" ) |  |  |  |
|  | | | | |  |
| # 6 | [**222,080**](https://apps.webofknowledge.com/summary.do?product=UA&doc=1&qid=111&SID=E1t3sem1IfBgUgnK7mF&search_mode=AdvancedSearch&update_back2search_link_param=yes) | TI=("social support*" or "group support*" or "group advice" or "group advis*" or "group monitor*" or "group intervention*" or "group train*" or "group instruct*" or "group consult*" or "group assist*" or "group educat*" or "group information" or "peer support*" or "peer advice" or "peer advis*" or "peer monitor*" or "peer intervention*" or "peer train*" or "peer instruct*" or "peer consult*" or "peer assist*" or "peer educat*" or "peer information" or "mutual support" or "telephone support" or "online support" or "virtual support" or "remote support" or "expert patient*" or "virtual communit*" or "online communit*" or befriend* or coach* or mentor or buddy or buddies or "health trainer*") OR AB=("social support*" or "group support*" or "group advice" or "group advis*" or "group monitor*" or "group intervention*" or "group train*" or "group instruct*" or "group consult*" or "group assist*" or "group educat*" or "group information" or "peer support*" or "peer advice" or "peer advis*" or "peer monitor*" or "peer intervention*" or "peer train*" or "peer instruct*" or "peer consult*" or "peer assist*" or "peer educat*" or "peer information" or "mutual support" or "telephone support" or "online support" or "virtual support" or "remote support" or "expert patient*" or "virtual communit*" or "online communit*" or befriend* or coach* or mentor or buddy or buddies or "health trainer*") |  |  |  |
|  | | | | |  |
| # 5 | [**462,114**](https://apps.webofknowledge.com/summary.do?product=UA&doc=1&qid=110&SID=E1t3sem1IfBgUgnK7mF&search_mode=CombineSearches&update_back2search_link_param=yes) | #4 OR #3 |  |  |  |
|  | | | | |  |
| # 4 | [**446,821**](https://apps.webofknowledge.com/summary.do?product=UA&doc=1&qid=107&SID=E1t3sem1IfBgUgnK7mF&search_mode=AdvancedSearch&update_back2search_link_param=yes) | TI=("self care" or selfcaring or self-care or "self caring" or "self manag*" or selfmanag* or self-manag* or selfassess* or "self assess*" or self-assess or selfdiagnos* or "self diagnos*" or self-diagnos* or selfreport* or self-report* or "self report*" or selfmonitor* or self-monitor* or "self monitor*" or self-policing or selfpolicing or "self policing" or self-supervis* or selfsupervis* or "self supervis*" or selfhelp* or self-help* or "self help*" or selftreat* or self-treat* or "self treat*" or selfmedicat* or self-medicat* or "self medicat*" or selfcure* or self-cure* or self-cure* or selfcuring or self-curing or "self curing" or selfrecover* or self-recover* or "self recover*" or "self initiat*" or self-initiat* or selfregulat* or self-regulat* or "self regulat*") OR AB=("self care" or selfcaring or self-care or "self caring" or "self manag*" or selfmanag* or self-manag* or selfassess* or "self assess*" or self-assess or selfdiagnos* or "self diagnos*" or self-diagnos* or selfreport* or self-report* or "self report*" or selfmonitor* or self-monitor* or "self monitor*" or self-policing or selfpolicing or "self policing" or self-supervis* or selfsupervis* or "self supervis*" or selfhelp* or self-help* or "self help*" or selftreat* or self-treat* or "self treat*" or selfmedicat* or self-medicat* or "self medicat*" or selfcure* or self-cure* or self-cure* or selfcuring or self-curing or "self curing" or selfrecover* or self-recover* or "self recover*" or "self initiat*" or self-initiat* or selfregulat* or self-regulat* or "self regulat*") |  |  |  |
|  | | | | |  |
| # 3 | [**16,577**](https://apps.webofknowledge.com/summary.do?product=UA&doc=1&qid=106&SID=E1t3sem1IfBgUgnK7mF&search_mode=AdvancedSearch&update_back2search_link_param=yes) | #1 NOT #2 |  |  |  |
|  | | | | |  |
| # 2 | [**40,079**](https://apps.webofknowledge.com/summary.do?product=UA&doc=1&qid=105&SID=E1t3sem1IfBgUgnK7mF&search_mode=AdvancedSearch&update_back2search_link_param=yes) | TI=(self-administer* near/2 (questionnaire* or survey* or interview*) ) OR AB=(self-administer* near/2 (questionnaire* or survey* or interview*) ) |  |  |  |
|  | | | | |  |
| # 1 | [**56,656**](https://apps.webofknowledge.com/summary.do?product=UA&doc=1&qid=104&SID=E1t3sem1IfBgUgnK7mF&search_mode=AdvancedSearch&update_back2search_link_param=yes) | TI=(self-administer*) OR AB=(self-administer*) |  |  |  |

**Database searched: Engineering Village**

**Date searched: 18 November 2021**

**Date range searched:**

**Records retrieved: 214**

**Search strategy:**

 ((("mobile health" or mHealth or m-Health or mhealth or m-health or e-health or e-Health or ehealth or eHealth or mcare or m-Care or mCare or cellphone or cellphones or "cell phones" or "cell phone" or "cell phones" or "cellular phone" or "cellular phones" or "cellular telephone" or "cellular telephones" or "mobile phone" or "mobile phones" or "mobile phone" or "mobile telephone" or "mobile telephones" or "handheld computer" or handheld computers" or "hand held computer" or "hand held computer" or smartphone or smartphones or tablets or tablets or ipad or ipads or iPad or iPads, or iphone or iphones or iPhone or iPhones or android or androids or blackberry or "wireless communication" or "wireless communications" or "wireless technology" or "wireless technologies" or "mobile communication" or "mobile communications" or "mobile application" or "mobile applications" or "mobile app" or "mobile apps" or software or "portable software application" or "portable mobile applications" or "portable mobile app" or "portable mobile apps" or pda or pdas or "personal digital assistant" or "mobile technology" or "mobile technologies" or "computer simulation" or "computer simulations" or "mobile game" or "mobile games" or "text messaging" or "test message" or texting or "short message service" or SMS or "reminder system" or "reminder systems" or electronic mail" or "electronic mailing" or "electronic mails" or email or emails or e-mail or e-mails or e-mailing or videorecording or "video recording" or "video recordings" or "video-recording" or "telemedicine" ot telecare or telemonitoring or teleheatlh or tele-health or "social media") WN ALL) AND (("sickle cell" or "sickle cell disease" or "sickle cell disorder" or "sickle cell syndrome" or "sickle cell anaemia" or "sickle cell anemia" or SCD or SCA or HbSS or HbSC or "haemoglobin S" or "hemoglobin S" or "haemoglobin SC" or "hemoglobin SC") WN ALL))
