# Supplementary material for: Self‐management interventions for children and young people with sickle cell disease: A systematic review
Source: Health Expect. 2023 Jan 3;26(2):579–612. doi: 10.1111/hex.13692 (PMC10010100; doi:10.1111/hex.13692)
Supplement: Supplementary file 2 — Supporting information. [file HEX-26--s002.docx]

**DATA EXTRACTION DOMAINS**

|  | **Excel Field Name (Ignore)** | **Field details and data extraction guidance** | **Enter data extracted in this column** |
| --- | --- | --- | --- |
|  | RM ID | **Unique ID** |  |
|  | Author | **Author details** |  |
|  | Date | **Date** |  |
|  | Extractor | **Person extracting the data** |  |
| **STUDY POPULATION** | Target | **Target population** |  |
|  | SCD diagnosis | **Diagnosis** |  |
|  | Inclusion | **Inclusion criteria** |  |
|  | Exclusion | **Exclusion criteria** |  |
|  | Sex | **Sex of the children/YP in the study at baseline** |  |
|  | Age | **Age of the children/YP in the study at baseline** |  |
|  | Ethnicity | **Ethnicity**  *Because of differences in how ethnicity is classified across countries, only salient features are required, e.g. 90% black* |  |
|  | Locale | **Locale**  *Provide details of the country where data was collected* |  |
|  | Recruitment | **Recruitment method**  *Brief description of actual recruitment method* |  |
| **STUDY AND INTERVENTION DESIGN** | Arms | **Arms**  *Number of groups, conditions or arms* |  |
|  | Control | **Description of the control**  *Provide a brief description* |  |
|  | Control type | **Type of control** |  |
|  | Blinding | **Type of blinding**  Indicate if participants, interventionists and/or assessors |  |
|  | Int Name | **Intervention name**  *Details of the intervention name (report full name as well as acronym if an acronym is used)* |  |
|  | Model | **Theoretical model** |  |
|  | Int | **Content** |  |
|  | Int development | **Model and PPI involvement**  *Detail the extent of PPI involvement* |  |
|  | Self-management | **Self-management element**  *Brief descriptions of the self-management element(s) that qualify the intervention as a self-management intervention* |  |
|  | Recipients | **Recipients** |  |
|  | Platform | **Platform/Delivery method(s)**  *Details of the platform/vehicle through which the intervention was delivered e.g., face-to-face, via the internet, telephone, printed manual* |  |
|  | Platform | **Location of int delivery**  *Detail the location for intervention delivery e.g., home, community, hospital, digital, internet-based or multiple* |  |
|  | Agent | **Self-management support agent**  *Details of the facilitator of the self care support e.g. nurse, clinical psychologist, peers* |  |
|  | Agent role | **Agent role**  *Role that the support agent had e.g. running groups, providing telephone support* |  |
|  | Agent trained? | **Did the support agent receive any special training for the role?**  *Answer yes, no or unclear. Note that this field is not asking if the agent was a trained professional but whether they were specially trained for the role.* |  |
|  | Agent supervised | **Support agent supervision**  *Details of any (clinical) supervision that the agent received* |  |
|  | Int Length | **Length of the intervention**  *To try and standardise this field, provide enough information for to work out average weekly contact time, e.g. 1 x 1 hour session per week over six weeks.* |  |
| **OUTCOMES** | Outcome | **Outcomes** |  |
|  | Principal outcome | **Principle outcome**  *Identified as the outcome that reflects the purpose of the study.* |  |
|  | Satisfaction/acceptability/engagement | **Is there any satisfaction data in the paper?**  *If yes, provide findings* |  |
|  | Feasibility | **Is there any feasibility data in the paper?**  *Provide details of the findings* |  |
|  | Implementation issues | **Is there any implementation data in the paper?**  *Provide details of the findings including attrition and retention rates* |  |
|  | Author’s conclusion | **Summarise author(s) conclusions** |  |
|  | Funding | **Indicate source of research funding** |  |
